# Discovery of Novel *α*-Aminophosphonates with Hydrazone as Potential Antiviral Agents Combined With Active Fragment and Molecular Docking

**DOI:** 10.3389/fchem.2022.911453

**Published:** 2022-05-20

**Authors:** Jia Tian, Renjing Ji, Huan Wang, Siyu Li, Guoping Zhang

**Affiliations:** ^1^ Chemistry and Material Science College, Huaibei Normal University, Huaibei, China; ^2^ Key Laboratory of Green and Precise Synthetic Chemistry and Applications, Ministry of Education, Huaibei Normal University, Huaibei, China

**Keywords:** *α*-aminophosphonate, hydrazone, synthesis, antiviral, docking

## Abstract

A series of novel *α-*aminophosphonate derivatives containing hydrazone were designed and synthesized based on active fragments. Bioassay results demonstrated that title compounds possessed good activities against tobacco mosaic virus. Among them, compounds 6a, 6g, 6i, and 6j were equivalent to the commercial antiviral agents like *dufulin*. On structure optimization-based molecular docking, compound 6k was synthesized and displayed excellent activity with values of 65.1% curative activity, 74.3% protective activity, and 94.3% inactivation activity, which were significantly superior to the commercial antiviral agents *dufulin* and *ningnanmycin.* Therefore, this study indicated that new lead compounds could be developed by adopting a joint strategy with active fragments and molecular docking.

## 1 Introduction

Tobacco mosaic virus (TMV) is one of the most widely studied plant viruses that can cause deformation and stunting of the leaves, flowers, and fruits of infected plants (Ritzenthaler, 2005). Plant diseases caused by tobacco mosaic virus (TMV) are difficult to control because TMV is absolutely parasitic, and transmissibility to host cells and plants may completely suppress immune system (Bos, 1982). Although several commercial antiviral agents against TMV have been used, efficient and practical varieties are few. The widely used antiviral agent ribavirin only gave less than 50% anti-TMV effect at 500 *μ*g/ml (Hansen and Stace Smith, 1989). The developing novel structure, remarkable effect, and environmentally friendly anti-TMV agents are needed urgently. At present, the main representative research groups are Wang Qingmin’s group and Song Baoan’s group on domestic development of anti-plant virus agents. In 2019–2021, Song Baoan’s group mainly designed and synthesized antiviral compounds based on active fragments through *in vitro* activity screening by microscale thermophoresis (MST) (He et al., 2019; Zan et al., 2020; Liu et al., 2021; Zhang et al., 2021). During the same period, Wang Qingming’s group mainly designed and synthesized new and efficient antiviral lead compounds based on natural products through traditional *in vivo* activity screening by using the half leaf dry spot method (Li et al., 2019; Hao et al., 2020a; Chen et al., 2020; Li et al., 2021). Then, the primary action mechanism of antiviral agents was studied by the *vivo* interaction of viral coat protein and drug molecules. These results indicated that viral coat protein was a key target protein for antiviral agents. Therefore, molecular docking could accelerate the development of antiviral agents.

**FIGURE 1:**
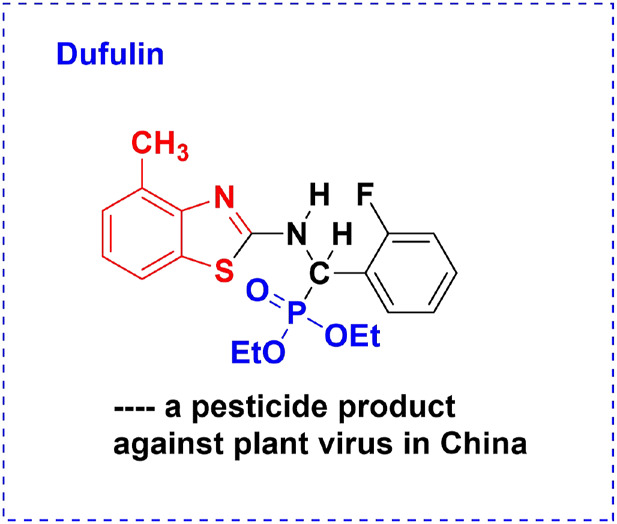
Structure of *dufulin*.

*α*-Aminophosphonates are considered to be structural analogs of *α*-amino acids. Compounds bearing the *α*-aminophosphonate moiety play an important role in biochemical and medicinal chemistry such as antitumor activity (Liu et al., 2010; Ye et al., 2014; Fang et al., 2016; Ewies et al., 2019; Zhang et al., 2020), antivirus activity (Zhang et al., 2010; Zhang and Liu, 2016; Zhang et al., 2017; Lan et al., 2017; Poola et al., 2020; Zhou et al., 2021), antimicrobial activity (He et al., 2015), and antibacterial activity (Dake et al., 2011; Hellal et al., 2017). In recent years, Song and coworkers (Chen et al., 2009; Yang et al., 2011; Zhang et al., 2016) reported that many *α*-aminophosphonates with anti-TMV and anti-CMV activity were synthesized via substructural splicing. Among them, dufulin (Figure 1), a new commercially registered plant antiviral product, was developed, which belongs to the *α*-aminophosphonate family (Song et al., 2006). In addition, it has been highly effective in preventing infection caused by rice viruses and tobacco mosaic virus and, as a result, has obtained a national invention patent in the People’s Republic of China. It was registered by the Ministry of Agriculture of China (LS 20071280, 20071282, and 20130359) and was subsequently industrialized for large-scale field application. It has been widely used to prevent and control rice, vegetable, and tobacco viral diseases in China. This may provide some useful information for the future design of novel structural aminophosphonates.

**FIGURE 2:**
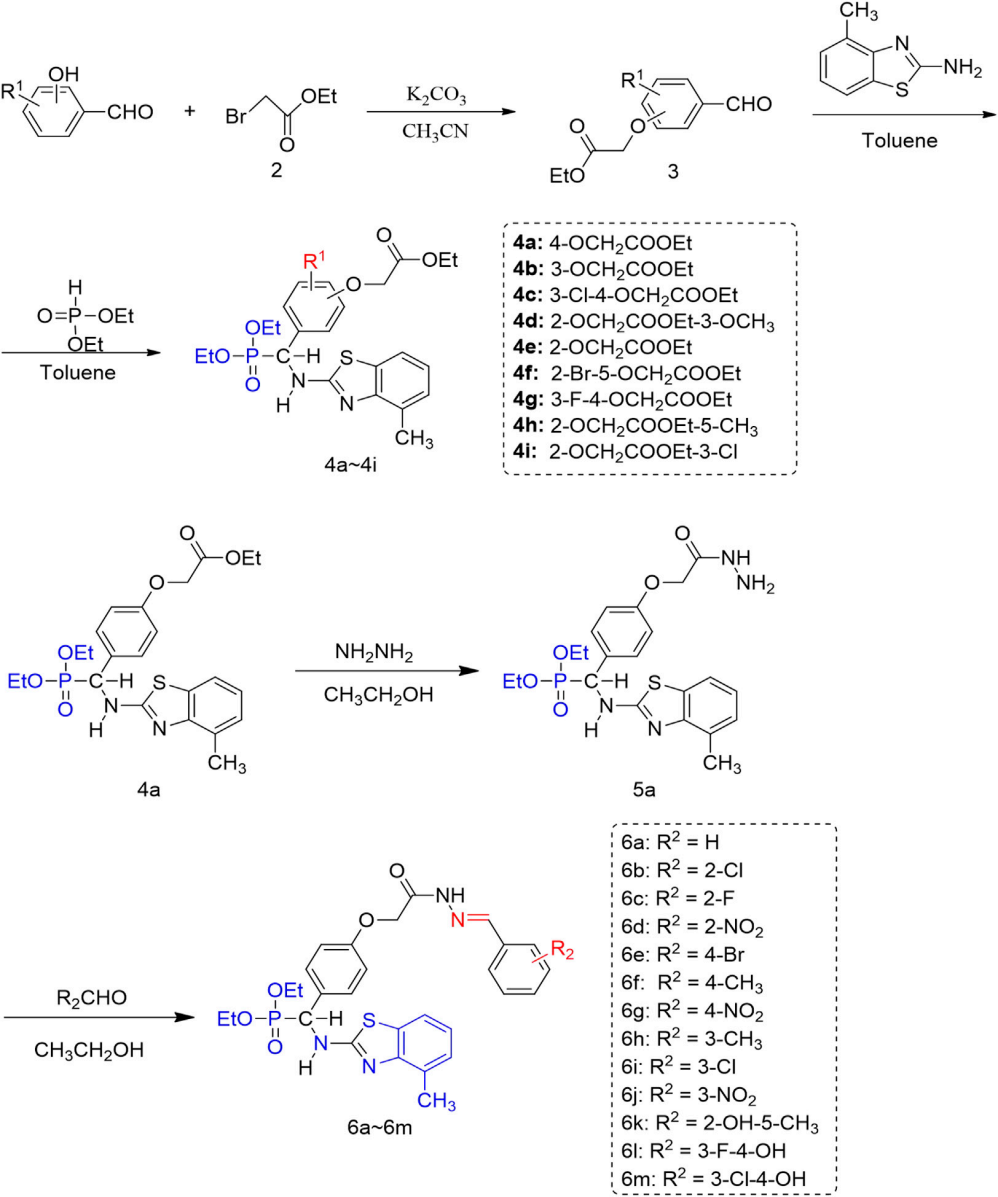
Synthetic routes of novel α-aminophosphonate derivatives with hydrazone.

Hydrazone derivatives are biologically interesting compounds known for their antiviral (Massarani et al., 1970; Wang et al., 2019), anticancer (Mehlika et al., 2012), insecticidal (Liu et al., 2010b), and antimicrobial (Martin et al., 2021) effects. Among them, hydrazones with promising antiviral activity have attracted our attention. In order to discover new molecules with antiviral effects, we sought to incorporate the active substructural unit hydrazone into the backbone structure of *α-*aminophosphonate. Based on the aforementioned facts, we designed and synthesized the title compounds by a joint strategy with active fragments and molecular docking (Figure 2). This article describes the syntheses and bioactivities of the designed compounds. The structure–activity relationships of these phosphonate–hydrazone analogs are examined in comparison with their parent aminophosphonate analogs to further the design of more effective antiviral compounds.

## 2 Materials and Methods

### 2.1 Chemicals

All reagents were purchased from commercial suppliers and used without further purification.

### 2.2 Instruments

^1^H NMR and ^13^C NMR spectra of the compounds were obtained using a Bruker DPX 400 MHz (Bruker, Germany) and Bruker DPX 600 MHzin CDCl3 or DMSO-*d*6 solution. HRMS was performed with a Thermo Scientific Q Exactive (Thermo Scientific, United States ). Infrared (IR) spectra were recorded on a Bruker VECTOR 22 spectrometer using KBr disks. The melting points of the compounds were measured using WRX-4 equipment.

### 2.3 General Procedures

#### 2.3.1 Procedures for the Synthesis of Intermediates (4a–4h)

Aromatic aldehyde containing a hydroxyl group (1, 100 mmol) was added to a vial containing acetonitrile (50 ml) and potassium carbonate (100 mmol), and then ethyl bromoacetate (2, 110 mmol) was added in the reaction vessel. After the mixture was stirred and refluxed for 12 h, the solvent was removed *in vacuo*. The reaction mixture was poured into water (100 ml) and extracted with dichloromethane (50 ml×3). The dichloromethane solution was dried with anhydrous Na_2_SO_4_ and evaporated in a vacuum. The residue was recrystallized from acetonitrile to obtain the intermediates (3a∼3 h). A solution of intermediates 3 (80 mmol) and 2-amino-4-methylbenzothiazole (80 mmol) in toluene (50 ml) was refluxed for 3 h. Then, diethyl phosphite (120 mmol) was added to the reaction solution and refluxed for 6–12 h. The crude product was afforded through removing the solvent and recrystallized from acetonitrile to obtain the intermediates (4a∼4 h). Characterization data of the intermediate 4a are given as follows, and the data of other compounds are listed in Supplementary Material S1.

4a: Yield 78%, m. p. 128–130°C; ^1^H NMR (600 MHz, DMSO) δ 8.89 (dd, J = 9.6, 2.9 Hz, 1H), 7.43 (dd, J = 14.9, 7.5 Hz, 3H), 7.01 (d, J = 7.3 Hz, 1H), 6.96–6.84 (m, 3H), 5.58 (dd, *J* = 21.0, 9.6 Hz, 1H), 4.73 (s, 2H), 4.12 (q, J = 7.1 Hz, 2H), 4.08–3.96 (m, 2H), 3.94–3.86 (m, 1H), 3.84–3.76 (m, 1H), 2.41 (s, 3H), 1.16 (t, J = 7.1 Hz, 3H), 1.13 (t, J = 7.0 Hz, 3H), 1.03 (t, J = 7.0 Hz, 3H). ^13^C NMR (151 MHz, DMSO) δ 169.13 (s), 164.99 (d, *J* = 9.7 Hz), 157.67 (s), 150.92 (s), 130.76 (s), 129.91 (d, *J* = 5.7 Hz), 129.01 (s), 128.00 (s), 126.68 (s), 121.69 (s), 118.86 (s), 114.75 (s), 65.13 (s), 63.07 (d, *J* = 6.7 Hz), 62.86 (d, *J* = 6.8 Hz), 61.09 (s), 54.84 (s), 53.81 (s), 18.41 (s), 16.72 (d, *J* = 5.4 Hz), 16.53 (d, *J* = 5.4 Hz), 14.48 (s). IR (thin film, cm^−1^): 3233.5 (s), 2982.6 (s), 2928.9 (s), 1753.3 (s), 1587.9 (s), 1534.9 (s), 1446.3 (s), 1197.7 (s), 1053.1 (s), 1018.7 (s), 976.1 (s). HRMS (ESI) m/z for (C_23_H_29_N_2_O_6_PS [M + H]^+^ cacld. 493.1557, found 493.1553.

#### 2.3.2 Procedures for the Synthesis of Intermediate 5a

The intermediate 4a (60 mmol) was added to the reaction flask with ethyl alcohol (50 ml), and then hydrazine hydrate (70 mmol) was added to the reaction mixture. After the mixture was stirred and refluxed for 12 h, ethyl alcohol was evaporated under reduced pressure to afford the crude product. The residue was purified by recrystallization affording acyl-hydrazine (5a) using alcohol. Characterization data of the intermediate 5a are given as follows.

5a: Yield 71%, m. p. 126–128°C; ^1^H NMR (600 MHz, DMSO-*d*_*6*_) *δ:* 9.29 (s, 1H), 8.92–8.85 (m, 1H), 7.49–7.38 (m, 3H), 7.01 (d, J = 7.4 Hz, 1H), 6.96–6.87 (m, 3H), 5.57 (dd, *J* = 21.0, 9.5 Hz, 1H), 4.44 (s, 2H), 4.32 (s, 2H), 4.08–3.95 (m, 2H), 3.93–3.87 (m, 1H), 3.83–3.77 (m, 1H), 2.40 (s, 3H), 1.13 (t, J = 7.0 Hz, 3H), 1.04 (t, J = 7.0 Hz, 3H); ^13^C NMR (151 MHz, DMSO-*d*_*6*_) *δ:* 167.03 (s), 165.00 (d, *J* = 9.8 Hz), 157.87 (s), 150.92 (s), 130.76 (s), 129.87 (d, *J* = 5.6 Hz), 128.94 (s), 127.99 (s), 126.68 (s), 121.69 (s), 118.87 (s), 114.88 (s), 66.74 (s), 63.07 (d, *J* = 6.7 Hz), 62.87 (d, *J* = 6.7 Hz), 56.50 (s), 54.89 (s), 53.86 (s), 19.03 (s), 18.41 (s), 16.73 (d, *J* = 5.2 Hz), 16.55 (d, *J* = 5.4 Hz). IR (thin film, cm^−1^): 3231.8 (s), 3037.2 (s), 2984.6 (s), 1671.9 (s), 1590.3 (s), 1536.9 (s), 1510.7 (s), 1446.6 (s), 1239.3 (s), 1050.8 (s), 1024.6 (s), 973.9 (s). HRMS (ESI) m/z for (C_21_H_27_N_4_O_5_PS [M + H]^+^ cacld. 479.1513, found. 479.1509.

#### 2.3.3 Procedures for the Synthesis of Title Compounds (6a∼6 m)

The intermediate 5a (2.0 mmol) and aldehyde (2.0 mmol) were added to 5 ml of alcohol. The mixture was stirred at 80°C for 6 h. The resulting mixture was concentrated under reduced pressure to give the crude product. A total of 5 ml of water was added to the crude product and stirred for 0.5 h. The crude product was purified by column chromatography using hexane/EtOAc (1:2, v/v) or recrystallization with alcohol. The data for the title compound 6a are shown as follows, and the data of other compounds are listed in Supplementary Material S1.

**6a**: Yield 86%, m. p. 184–186°C; ^1^H NMR (400 MHz, DMSO-*d*_*6*_) *δ:* 11.56 (trans), 11.51 (cis) (s, 1H, CONH), 8.90 (d, *J* = 9.6 Hz, 1H, NH-Hetero), 8.30 (cis), 7.97(trans) (s, 1H, CH = N), 7.74–7.60 (m, 2H, Ar-H), 7.47–7.36 (m, 6H, Ar-H), 7.07–6.84 (m, 4H, Ar-H), 5.58 (dd, *J* = 21.0, 9.6 Hz, 1H, CHP), 5.10 (trans), 4.63(cis) (s, 2H, COCH_2_O), 4.10–3.96 (m, 2H, CH_2_OP), 3.95–3.87 (m, 1H, CHOP), 3.86–3.75 (m, 1H, CHOP), 2.41 (s, 3H, CH_3_-Hetero), 1.13 (t, *J* = 7.0 Hz, 3H, CH_3_), 1.04 (t, *J* = 7.0 Hz, 3H, CH_3_). trans:cis=(0.61:0.39); ^13^C NMR (151 MHz, DMSO-*d*_*6*_) *δ:* 169.03 (s), 164.61 (d, *J* = 9.6 Hz), 164.25 (s), 157.89 (s), 157.43 (s), 150.55 (s), 148.04 (s), 143.86 (s), 134.18 (s), 134.04 (s), 130.37 (s), 130.24 (s), 130.01 (s), 129.56 (d, *J* = 5.3 Hz), 129.44 (d, *J* = 5.3 Hz), 128.88 (d, *J* = 4.6 Hz), 128.72 (s), 128.12 (s), 127.60 (s), 127.21 (s), 127.00 (s), 126.28 (s), 121.28 (s), 118.47 (s), 114.54 (s), 114.35 (s), 66.63 (s), 64.84 (s), 62.66 (d, *J* = 6.9 Hz), 62.46 (d, *J* = 6.5 Hz), 54.48 (s), 53.45 (s), 18.04 (s), 16.34 (d, *J* = 5.2 Hz), 16.16 (d, *J* = 5.3 Hz). IR (thin film, cm^−1^): 3273.9 (s), 3106.5 (s), 2979.7 (s), 2914.9 (s), 1695.5 (s), 1612.8 (s), 1586.6 (s), 1534.4 (s), 1511.5 (s), 1430.5 (s), 1229.8 (s), 1047.5 (s), 1023.7 (s); HRMS (ESI) m/z for (C_28_H_31_N_4_O_5_PS [M + H]^+^ cacld. 567.1826, found. 567.1824.

#### 2.3.4 Evaluation of Phytotoxic Activities Against Tobacco and Anti-TMV Activity

Phytotoxic activities against tobacco and anti-TMV activity were assessed according to the aforementioned method (Ji et al., 2019; Wang et al., 2012). Tobacco mosaic virus (TMV) was purified. The biological activity of the compounds against TMV was evaluated by using a half-leaf method.

### 2.4 Molecular Docking

Molecular docking with AutoDock 4.0 (Huey et al., 2007; Trott and Olson, 2010) between compound 6a and TMV-CP was performed. The X-ray crystal structure of TMV-CP used for the computation was downloaded from RCSB (Bhyravbhatla et al., 1998). Most of the parameters for the docking calculation were set to the default values. Each docked structure was scored by the built-in scoring function and was clustered by 1 Å of RMSD criteria. Finally, the enzyme ligand complex structures were selected according to the criteria for autodocking score.

## 3 Results and Discussion

### 3.1 Synthesis and Spectroscopy

The synthetic route of the target compounds (6a∼6 m) is shown in Figure 2. 4-hydroxybenzaldehyde 1) reacted with ethyl bromoacetate, 2) in acetonitrile to give aldehyde with an ester group, and 3) compound 3 condensed with 2-amino-4-methylbenzothiazole via a Schiff base condensation to give the intermediate of imine, which was followed by phosphine hydrogenation with diethyl phosphite to afford the compound 4. Compound 4 continued to react with hydrazine hydrate in alcohol to give the intermediate compound 5. Title compound 6 was smoothly prepared by the reaction of an aromatic aldehyde with the corresponding hydrazides. The chemical structures of these compounds were identified by NMR, IR, and HRMS (Supplementary Material). In ^1^H NMR of the title compounds, the CH-P proton appeared at δ 5.58–5.63 as dd, the NH-Ar proton appeared at δ 8.89–8.93 as double, and the CH = N proton of *cis*-isomer appeared at the higher magnetic field (8.17–8.70) than *trans*-isomer (7.85–8.34), the ratio of which was 3:2. In the ^13^C NMR spectra of compound 6, the typical carbon resonance at δ169 was indicative of a carbonyl group (C═O). The IR spectra of compound C showed bands at 1684–1701 cm^−1^ for C═O stretching.

### 3.2 Phytotoxic Activities and Antivirus Activities

First, the data on phytotoxic activity against tobacco indicated that compounds 6a–6k at 500 *μ*g·mL^−^ showed no toxicity. Then, the tested concentration of the compounds at 500 *μ*g/ml was chosen, and the biological activities against TMV were evaluated. The results of anti-TMV activity are shown in Table1. The intermediates (4a∼4h) exhibited lower antiviral activities against TMV *in vivo.* Among them, the intermediate 4a displayed moderate activities, with values of 33.2% curative activity, 45.7% protective activity, and 78.7% inactivation activity. The intermediate 5a exhibited higher activities than compound 4a, especially in curative activity. The title compounds 6a, 6g, 6i, and 6j derived from *α-*aminophosphonate possessed good activities, which were similar to those controls of *ningnanmycin* and *dufulin.* Compound 6k exhibited excellent activity, with the values of 65.1% curative activity, 74.3% protective activity, and 94.3% inactivation activity, which were significantly greater than those of controls. Compounds 6b, 6f, and 6h possessed slightly lower activity than these controls. Other compounds showed lower activities. The antiviral activity results of the intermediates 4a∼4i and intermediate 5a suggest that the structure of *α-*aminophosphonate with benzothiazole is critical for the activity, and generally, in addition, the R_2_ substituted phenyl series of the title compounds influenced the antiviral activity for the derivatives (6a∼6 m). The R_2_ substitutions at the ortho-position of the phenyl ring connected to the hydrazone moiety (6b, 6c, and 6d) showed weaker curative activity than the R_2_ = H of the phenyl ring. As for the para-substituted derivatives, it is clear to see that the order of the curative activity against TMV was NO_2_-substituted (6g) > CH_3_-substituted (6f) > Br-substituted (6e). When the R_2_ group was at the meta-position of the phenyl ring, the compounds (6h∼6j) exhibited relatively similar curative activities to compound 6a (R_2_ = H). Fortunately, 2-OH-5-CH_3_-substituted compound **6k** showed the best curative activity (65.1%) against TMV, which was significantly better than that of *ningnanmycin* (53.3%). Considering the prior discussion, we found that the antiviral activities of our designed compounds could be increased by the introduction of 2-OH and 5-CH_3_ on benzene rings. The results further suggested that small differences of substituted position on the phenyl ring could lead to large differences in the overall activities, which implies further possibilities for lead compound development.

**TABLE 1:** Antiviral activity of the compounds (4a∼6m) against TMV at 500 *μ*g/mL^a^.

Compound	Curative activity^a^ (%)	Protective activity^a^ (%)	Inactivation activity^a^ (%)
4a	33.2 ± 2.6	45.7 ± 2.1	78.7 ± 2.2
4b	21.2 ± 3.1	35.6 ± 2.7	48.6 ± 1.8
4c	26.3 ± 3.3	37.1 ± 2.6	52.2 ± 2.4
4d	0	9.1 ± 3.4	0
4e	0	0	0
4f	23.6 ± 2.8	43.3 ± 2.1	57.4 ± 2.3
4g	27.5 ± 3.2	44.1 ± 1.6	67.6 ± 2.1
4 h	22.2 ± 2.4	34.6 ± 1.9	51.3 ± 2.8
5a	50.3 ± 1.6	49.7 ± 1.9	83.1 ± 2.6
6a	51.3 ± 1.4	54.1 ± 1.7	90.3 ± 1.3
6b	47.6 ± 2.1	35.8 ± 3.3	49.3 ± 2.7
6c	41.6 ± 1.9	34.8 ± 3.5	51.3 ± 3.1
6d	39.1 ± 2.8	53.3 ± 1.4	77.6 ± 1.9
6e	29.3 ± 3.6	32.5 ± 2.7	45.3 ± 2.4
6f	48.6 ± 2.3	31.3 ± 3.1	43.1 ± 2.9
6g	56.7 ± 1.3	38.5 ± 2.9	49.3 ± 2.6
6 h	48.1 ± 2.1	45.0 ± 2.3	77.3 ± 2.3
6i	54.2 ± 1.8	56.6 ± 1.3	81.5 ± 1.7
6j	55.5 ± 1.8	60.0 ± 1.2	88.7 ± 2.7
6k	65.1 ± 1.2	74.3 ± 1.5	94.3 ± 1.1
6L	32.2 ± 3.3	35.4 ± 3.7	60.1 ± 1.8
6 m	31.4 ± 2.9	33.7 ± 3.4	57.3 ± 2.1
*Dufulin* *^b^*	50.3 ± 2.6	54.3 ± 1.9	87.6 ± 2.2
*Ningnanmycin* *^c^*	53.3 ± 2.3	58.3 ± 1.7	92.3 ± 1.4

a Average of three replicates.

b *Dufulin* was also used as the control.

c *Ningnanmycin* was used as the control.

### 3.3 Molecular Modeling Analysis

**FIGURE 3:**
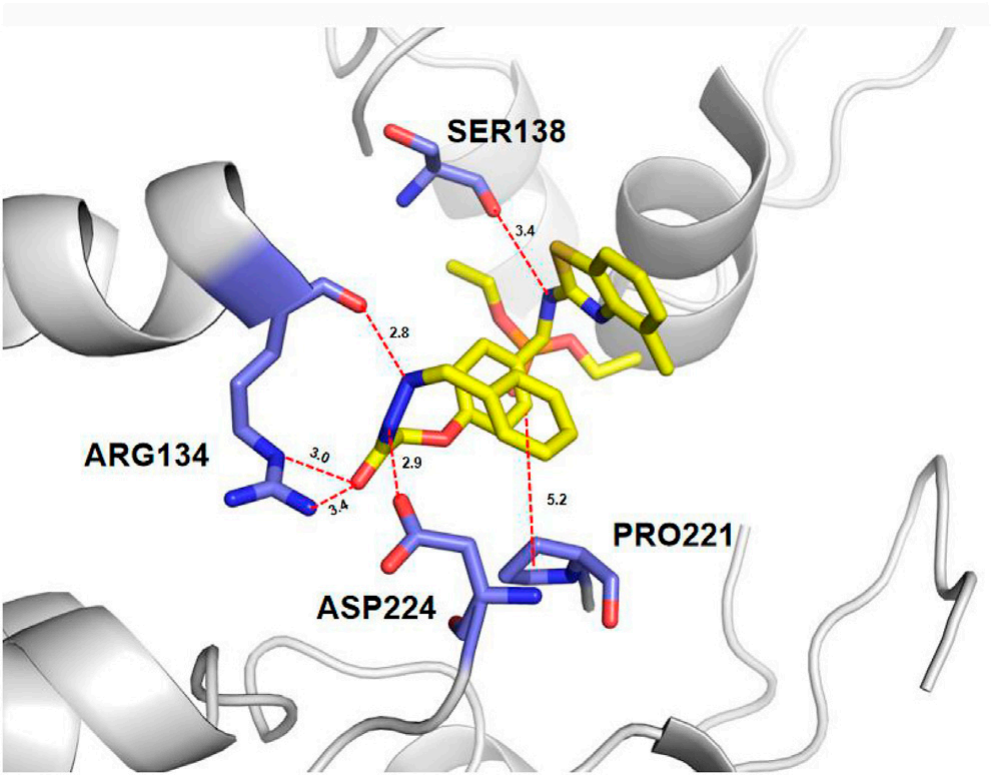
3D mode of the interaction of compound 6a and receptor TMV-CP analyzed by AutoDock 4.0 software. Conventional hydrogen bond, carbon–hydrogen bond, and alkyl, as well as Pi–alkyl, are shown by green, light green, and pink, respectively.

TMV-CP is an important protein involved in plant virus infections and is being studied as a potential protein target to develop effective antivirus agents (Hao et al., 2020b; Kang et al., 2020). In order to gain more understanding of the structure–activity relationships and further structure optimization, molecular docking was performed on the binding mode of compound 6a into the binding pocket of TMV-CP using AutoDock 4.0 software. The 3D binding models of compound 6a with TMV-CP are shown in Figure 3. Results showed that the benzothiazole ring of 6a fit into the binding pocket, surrounded by the amino acid residues of SER138. Detailed analysis of the binding mode showed that the hydrazone of 6a was surrounded by the amino acid residues of ARG134 and ASP224. These docking results suggested that the benzothiazole ring in the title compound was critical for the activity and the phenyl ring of hydrazone influenced the antiviral activity. On structure optimization-based molecular docking, compound 6k was synthesized and displayed excellent activity, with values of 65.1% curative activity, 74.3% protective activity, and 94.3% inactivation activity, which were significantly superior to the commercial antiviral agents *dufulin* and *ningnanmycin.* Therefore, molecular docking could accelerate the development of lead compounds.

## 4 Conclusion

A series of *α-*aminophosphonate-hydrazone derivatives were synthesized and evaluated for antiviral activities. Some derivatives showed good inhibitory activity. The SARS analysis showed that the volume and position of the substituted groups at the phenyl ring of hydrazones had significant influences on inhibitory activity. The docking studies showed that compound **6a** was well bound to the TMV-CP via one hydrogen bond with SER 138, ARG 134, and ASP 224. This also indicated that the structures of benzothiazole and hydrazone play a key role in the activity of the title compound. Among them, compound 6k displayed excellent activity, with values of 65.1% curative activity, 74.3% protective activity, and 94.3% inactivation activity. Therefore, the basic motif of compound 6k can be used as a lead compound for further development.

## Data Availability

The original contributions presented in the study are included in the article/Supplementary Material, further inquiries can be directed to the corresponding author.
