# Supplementary material for: Discovery of Novel α-Aminophosphonates with Hydrazone as Potential Antiviral Agents Combined With Active Fragment and Molecular Docking
Source: Front Chem. 2022 May 20;10:911453. doi: 10.3389/fchem.2022.911453 (PMC10588822; doi:10.3389/fchem.2022.911453)
Supplement: Supplementary file 1 [file DataSheet1.pdf]

Supplementary Information for

Discovery of Novel α-Aminophosphonate-Hydrazones as Potential Antiviral Agents Combined with Active Fragment and Molecular Docking

JiaTian [&], Renjing Ji [&], Huan Wang, Siyu Li, Guoping Zhang [*]

[1]Key Laboratory of Green and Precise Synthetic Chemistry and Applications, Ministry of Education，Huaibei Normal University, Huaibei 235000, China

[2]Chemistry and Material Science College, Huaibei Normal University, Huaibei 235000, China

Table of Contents

(a) Characterization data of intermediates **4a-4h**……………………….

(b) Characterization data of the title compounds **6a-6m**………………

(c) ^{1}H and ^{13}C NMR Spectra of compounds 6a~6m………………

1. Characterization data of intermediates (4a-4h)

4a: 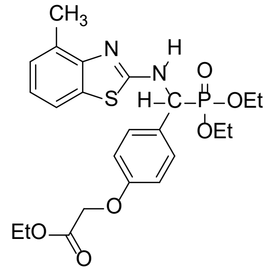

Yield78%, m.p. 128-130℃; ^{1}H NMR (600 MHz, DMSO) δ 8.89 (dd, J = 9.6, 2.9 Hz, 1H), 7.43 (dd, J = 14.9, 7.5 Hz, 3H), 7.01 (d, J = 7.3 Hz, 1H), 6.96 – 6.84 (m, 3H), 5.58 (dd, J = 21.0, 9.6 Hz, 1H), 4.73 (s, 2H), 4.12 (q, J = 7.1 Hz, 2H), 4.08 – 3.96 (m, 2H), 3.94 – 3.86 (m, 1H), 3.84 – 3.76 (m, 1H), 2.41 (s, 3H), 1.16 (t, J = 7.1 Hz, 3H), 1.13 (t, J = 7.0 Hz, 3H), 1.03 (t, J = 7.0 Hz, 3H). ^{13}C NMR (151 MHz, DMSO) δ 169.13 (s), 164.99 (d, *J* = 9.7 Hz), 157.67 (s), 150.92 (s), 130.76 (s), 129.91 (d, *J* = 5.7 Hz), 129.01 (s), 128.00 (s), 126.68 (s), 121.69 (s), 118.86 (s), 114.75 (s), 65.13 (s), 63.07 (d, *J* = 6.7 Hz), 62.86 (d, *J* = 6.8 Hz), 61.09 (s), 54.84 (s), 53.81 (s), 18.41 (s), 16.72 (d, *J* = 5.4 Hz), 16.53 (d, *J* = 5.4 Hz), 14.48 (s). IR (thin film, cm^{-1}): 3233.5 (s), 2982.6 (s), 2928.9 (s), 1753.3 (s), 1587.9 (s), 1534.9 (s), 1446.3 (s), 1197.7 (s), 1053.1 (s), 1018.7 (s), 976.1 (s). HRMS (ESI) m/z for ($C_{23}H_{29}N_2O_6PS$ $[M+H]^+$ cacld. 493.1557, found.493.1553. Anal. Calcd for $C_{23}H_{29}N_2O_6PS$: C, 56.09; H, 5.93; N, 5.69; Found: C, 56.10; H, 5.92; N, 5.71.

4b: 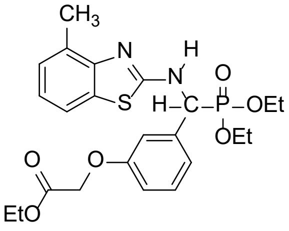

Yield 83%, m.p. 119-121℃; ^{1}H NMR (600 MHz, DMSO) δ 8.92 (d, *J* = 8.4 Hz, 1H), 7.45 (d, *J* = 7.7 Hz, 1H), 7.26 (t, *J* = 8.0 Hz, 1H), 7.10 (d, *J* = 6.3 Hz, 2H), 7.02 (d, *J* = 7.3 Hz, 1H), 6.90 (t, *J* = 7.6 Hz, 1H), 6.83 (d, *J* = 8.0 Hz, 1H), 5.63 (dd, *J* = 21.3, 9.6 Hz, 1H), 4.74 (s, 2H), 4.12 (q, *J* = 7.0 Hz, 2H), 4.07 – 3.96 (m, 2H), 3.94 – 3.87 (m, 1H), 3.85 – 3.77 (m, 1H), 2.40 (s, 3H), 1.16 (t, *J* = 7.1 Hz, 3H), 1.13 (t, *J* = 7.0 Hz, 3H), 1.04 (t, *J* = 7.0 Hz, 3H); ^{13}C NMR (151 MHz, DMSO) δ 168.60 (s), 164.53 (d, *J* = 10.6 Hz), 157.53 (s), 150.42 (s), 137.51 (s), 130.31(s), 129.27 (s), 127.59 (s), 126.24 (s), 121.29 (s), 118.42(s) , 114.73(d, *J* = 6.0 Hz), 113.44 (s), 64.65, 62.70 (d, *J*

= 6.8 Hz), 62.52 (d, J = 6.8 Hz), 60.64 (s), 54.43 (d, J = 154.0 Hz), 17.93 (s), 16.24 (d, J = 5.3 Hz), 16.05 (d, J = 5.3 Hz), 14.01 (s); IR (thin film, cm^{-1}): 3261.9 (s), 2982.8 (s), 2930.4 (s) , 1757.2 (s), 1588.3 (s), 1535.6 (s), 1486.8 (s) , 1234.9 (s), 1207.7 (s) , 1055.3 (s) , 1018.5 (s), 983.4 (s). HRMS (ESI) m/z for ($C_{23}H_{29}N_2O_6PS$ $[M+H]^+$ cacld. 493.1557, found. 493.1555.

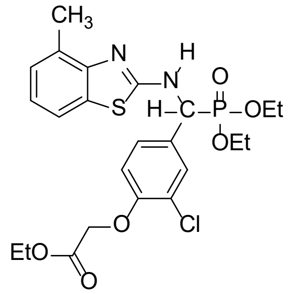

4c:

Yield 86%, m.p. 140-142°C; ^{1}H NMR (600 MHz, DMSO) δ 8.91 (d, J = 9.3 Hz, 1H), 7.60 (s, 1H), 7.46 (d, J = 7.8 Hz, 1H), 7.39 (d, J = 8.5 Hz, 1H), 7.03 (dd, J = 12.3, 8.2 Hz, 2H), 6.91 (t, J = 7.6 Hz, 1H), 5.59 (dd, J = 21.0, 9.4 Hz, 1H), 4.88 (s, 2H), 4.19 – 4.09 (m, 2H), 4.09 – 3.98 (m, 2H), 3.97 – 3.91 (m, 1H), 3.89 – 3.81 (m, 1H), 2.41 (s, 3H), 1.15 (dt, J = 14.0, 7.0 Hz, 6H), 1.05 (t, J = 7.0 Hz, 3H); ^{13}C NMR (151 MHz, DMSO) δ 168.66 (s), 164.88 (d, J = 10.6 Hz) (s), 164.84 (s), 153.04 (s), 150.85 (s), 130.79 (s), 130.30 (s), 130.12 (d, J = 5.2 Hz), 128.53 (d, J = 5.7 Hz), 128.13 (s), 126.72 (s), 121.83 (s), 121.47 (s), 118.91 (s), 113.94 (s), 65.69 (s), 62.13 (dd, J = 37.8, 6.8 Hz), 61.24 (s), 53.90 (d, J = 155.5 Hz), 18.38 (s), 16.60 (dd, J = 27.2, 5.4 Hz), 14.45 (s). IR (thin film, cm^{-1}): 3237.5 (s), 3034.6 (s), 2991.3 (s), 1758.1 (s), 1592.3 (s), 1541.3 (s), 1499.7 (s), 1233.2 (s), 1209.3 (s), 1052.6 (s), 1023.8 (s), 982.4 (s). HRMS (ESI) m/z for ($C_{23}H_{28}ClN_2O_6PS$ $[M+H]^+$ cacld. 527.1167, found. 527.1163.

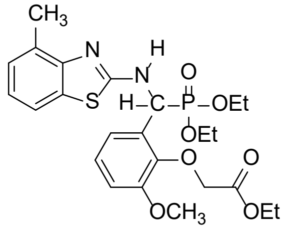

4d:

Yield 73%, m.p. 183-185 °C; ^{1}H NMR (600 MHz, DMSO) δ 8.86 (d, J = 9.6 Hz, 1H), 7.44 (d, J = 7.8 Hz, 1H), 7.18 – 7.03 (m, 2H), 6.99 (t, J = 9.7 Hz, 2H), 6.89 (t, J = 7.5 Hz, 1H), 6.22 (dd, J = 21.6, 9.7 Hz, 1H), 4.69 (dd, J = 47.8, 15.3 Hz, 2H), 4.23 – 4.14 (m, 2H), 4.12 – 4.02 (m, 2H), 3.99 – 3.91 (m, 1H), 3.88 – 3.80 (m, 1H), 3.77 (s, 3H), 2.34 (s, 3H), 1.21 (t, J = 7.0 Hz, 3H), 1.16

(t, J = 7.0 Hz, 3H), 1.04 (t, J = 7.0 Hz, 3H); ^{13}C NMR (151 MHz, DMSO) δ 169.11 (s), 165.15 (d, J = 10.8 Hz), 152.15 (s), 150.93 (s), 144.95 (d, J = 6.7 Hz), 130.76 (s), 130.32 (s), 127.93 (s), 126.71 (s), 124.83 (s), 121.73 (s), 120.69 (s), 118.87 (s), 112.84 (s), 69.64 (s), 62.91 (dd, J = 33.2, 6.7 Hz), 60.91 (s), 56.30 (s), 48.18 (d, J = 155.5 Hz), 18.38 (s), 16.61 (dd, J = 37.8, 5.4 Hz), 14.51 (s). IR (thin film, cm^{-1}): 3218.3 (s), 3022.8 (s), 2976.3 (s), 1752.6 (s), 1586.6 (s), 1532.4 (s), 1479.6 (s), 1242.1 (s), 1212.8 (s), 1052.0 (s), 1023.2 (s), 980.8 (s). HRMS (ESI) m/z for ($C_{24}H_{31}N_2O_7PS$ [M+H]$^+$ cacld. 523.1662, found. 523.1659.

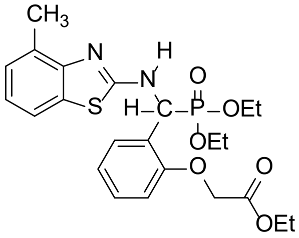

4e:

Yield 87%, m.p. 156-158 °C; ^{1}H NMR (600 MHz, DMSO) δ 8.86 (d, J = 8.2 Hz, 1H), 7.55 (d, J = 7.5 Hz, 1H), 7.44 (d, J = 7.7 Hz, 1H), 7.23 (t, J = 7.6 Hz, 1H), 7.00 (d, J = 5.9 Hz, 2H), 6.93 (d, J = 8.2 Hz, 1H), 6.89 (t, J = 7.5 Hz, 1H), 6.28 (dd, J = 20.7, 6.9 Hz, 1H), 4.81 (s, 2H), 4.17 (q, J = 7.0 Hz, 2H), 4.12 – 4.00 (m, 2H), 3.92 – 3.84 (m, 1H), 3.83 – 3.72 (m, 1H), 2.38 (s, 3H), 1.20 (t, J = 7.1 Hz, 3H), 1.15 (t, J = 7.0 Hz, 3H), 1.01 (t, J = 7.0 Hz, 3H); ^{13}C NMR (151 MHz, DMSO) δ 168.99 (s), 164.98 (d, J = 9.7 Hz), 155.66 (d, J = 6.4 Hz), 151.02 (s), 130.83 (s), 129.48 (d, J = 3.4 Hz), 129.44 (d, J = 3.0 Hz), 128.02 (s), 126.63 (s), 125.70 (s), 121.83 (s), 121.63 (s), 118.81 (s), 113.31 (s), 66.63 (s), 62.91 (dd, J = 18.7, 6.8 Hz), 61.13 (s), 47.82 (d, J = 157.0 Hz), 18.22 (s), 16.59 (dd, J = 37.8, 5.4 Hz), 14.50 (s). IR (thin film, cm^{-1}): 3238.8 (s), 3045.7 (s), 2979.8 (s), 1757.8 (s), 1595.2 (s), 1537.7 (s), 1451.0 (s), 1276.1 (s), 1201.9 (s), 1056.4 (s), 1028.7 (s), 981.3 (s). HRMS (ESI) m/z for ($C_{23}H_{29}N_2O_6PS$ [M+H]$^+$ cacld. 493.1557, found. 493.1551.

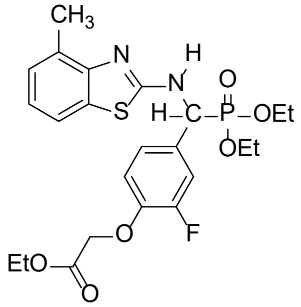

4f:

Yield 75%, m.p. 140-142°C; ^{1}H NMR (600 MHz, DMSO) δ 8.90 (d, J = 8.4 Hz, 1H), 7.46 (d, J = 7.7 Hz, 1H), 7.38 (d, J = 12.3 Hz, 1H), 7.23 (d, J = 8.3 Hz, 1H), 7.08 (t, J = 8.6 Hz, 1H), 7.02

(d, J = 7.3 Hz, 1H), 6.91 (t, J = 7.5 Hz, 1H), 5.59 (dd, J = 21.0, 9.5 Hz, 1H), 4.84 (s, 2H), 4.12 (q, J = 7.0 Hz, 2H), 4.09 – 3.97 (m, 2H), 3.96 – 3.90 (m, 1H), 3.89 – 3.78 (m, 1H), 2.41 (s, 3H), 1.30 – 1.09 (m, 6H), 1.05 (t, J = 7.0 Hz, 3H); ^{13}C NMR (151 MHz, DMSO) δ 168.76 (s), 164.90 (d, J = 10.1 Hz), 151.52 (d, J = 243.1Hz), 150.86(s), 145.48 (d, J = 10.2 Hz), 130.78 (s), 130.10 (d, J = 5.7 Hz), 128.12 (s), 126.72 (s), 124.91 (q, J = 3.0 Hz), 121.82 (s), 118.91 (s), 116.39 (dd, J = 19.1,4.5 Hz), 115.16 (s), 65.71 (s), 63.11 (dd, J = 19.6, 6.8 Hz), 61.21 (s), 54.03 (d, J = 155.5 Hz), 18.39 (s), 16.61 (dd, J = 27.2, 5.4 Hz), 14.45 (s). IR (thin film, cm^{-1}): 3234.9 (s), 2986.3 (s), 2932.7 (s), 1753.7 (s), 1588.9 (s), 1534.9 (s), 1440.0 (s), 1236.2 (s), 1201.2 (s), 1052.1 (s), 1017.9 (s), 969.4 (s). HRMS (ESI) m/z for ($C_{23}H_{28}FN_2O_6PS$ $[M+H]^+$ cacld. 511.1463, found. 511.1466.

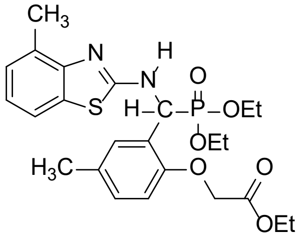

4g:

Yield 68%, m.p. 139-141°C; ^{1}H NMR (600 MHz, DMSO) δ 8.81 (d, J = 9.4 Hz, 1H), 7.44 (d, J = 7.7 Hz, 1H), 7.36 (s, 1H), 7.02 (dd, J = 17.4, 7.8 Hz, 2H), 6.89 (t, J = 7.6 Hz, 1H), 6.83 (d, J = 8.3 Hz, 1H), 6.24 (dd, J = 20.8, 9.6 Hz, 1H), 4.76 (s, 2H), 4.17 (q, J = 7.0 Hz, 2H), 4.14 – 4.00 (m, 2H), 3.93 – 3.85 (m, 1H), 3.83 – 3.74 (m, 1H), 2.38 (s, 3H), 2.22 (s, 3H), 1.20 (t, J = 7.1 Hz, 3H), 1.15 (t, J = 7.0 Hz, 3H), 1.02 (t, J = 7.0 Hz, 3H); ^{13}C NMR (151 MHz, DMSO) δ 169.10 (s), 164.96 (d, J = 9.6 Hz), 153.68 (d, J = 6.4 Hz), 151.04 (s), 130.83 (s), 130.61 (s), 129.91 (d, J =4.6 Hz), 129.73 (s), 128.00 (s), 126.63 (s), 125.44 (s), 121.62 (s), 118.81 (s), 113.53 (s), 66.95 (s), 62.90 (dd, J = 20.1, 6.8 Hz), 61.08 (s), 47.80 (d, J = 155.5 Hz), 20.80 (s), 18.23 (s), 16.58 (dd, J =34.7, 5.4 Hz), 14.50 (s). IR (thin film, cm^{-1}): 3245.4 (s), 3040.6 (s), 2979.4 (s), 1750.3 (s), 1586.2 (s), 1541.0 (s), 1503.8 (s), 1441.5(s), 1233.8 (s), 1200.1 (s), 1051.1 (s), 1028.8 (s), 973.6(s). HRMS (ESI) m/z for ($C_{24}H_{31}N_2O_6PS$ $[M+H]^+$ cacld. 507.1713, found. 507.1711.

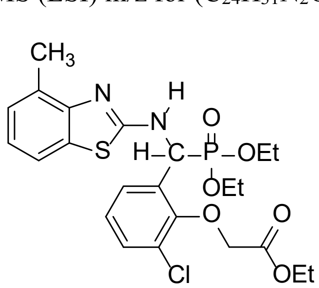

4h:

Yield 81%, m.p. 165-167 °C; ^{1}H NMR (600 MHz, DMSO) δ 9.07 – 8.97 (m, 1H), 7.56 (d, J

= 7.6 Hz, 1H), 7.52 – 7.42 (m, 2H), 7.26 (t, *J* = 7.5 Hz, 1H), 7.04 (d, *J* = 6.9 Hz, 1H), 6.94 (t, *J* = 7.1 Hz, 1H), 6.19 (dd, *J* = 21.9, 9.3 Hz, 1H), 5.03 (d, *J* = 15.1 Hz, 1H), 4.73 (d, *J* = 15.0 Hz, 1H), 4.28 – 4.19 (m, 2H), 4.17 – 4.10 (m, 2H), 4.04 – 3.97 (m, 1H), 3.94 – 3.87 (m, 1H), 2.37 (s, 3H), 1.23 (dt, *J* = 13.3, 6.4 Hz, 6H), 1.08 (t, *J* = 6.5 Hz, 3H); ^{13}C NMR (151 MHz, DMSO) δ 168.32 (s), 165.12 (d, *J* = 11.2 Hz), 151.95 (d, *J* = 6.5 Hz), 150.77 (s), 132.73 (s), 130.77 (s), 130.43 (s), 128.33 (s), 128.07 (s), 127.10 (s), 126.79 (s), 126.35 (s), 121.92 (s), 118.94 (s), 70.02 (s), 63.41 (d, *J* = 6.8 Hz), 63.16 (d, *J* = 6.7 Hz), 61.24 (s), 49.07 (s), 48.05 (s), 18.47 (s), 16.70 (d, *J* = 5.2 Hz), 16.42 (d, *J* = 5.4 Hz), 14.45 (s). IR (thin film, cm^{-1}): 3217.8 (s), 3010.2 (s), 2980.4 (s), 1756.5 (s), 1585.9 (s), 1531.2 (s), 1449.1 (s), 1241.4 (s), 1209.9 (s), 1049.8 (s), 1025.6 (s), 984.3 (s). HRMS (ESI) m/z for ($C_{23}H_{28}ClN_2O_6PS$ $[M+H]^+$ cacld. 527.1167, found. 527.1163.

2. Characterization data of the title compounds (6a~6m)

6a: Yield 86%, m.p. 184-186 ^{0}C; ^{1}H NMR (400 MHz, DMSO) δ 11.56 (trans), 11.51 (cis) (s, 1H, CONH), 8.90 (d, *J* = 9.6 Hz, 1H, NH-Ar), 8.30 (cis), 7.97(trans) (s, 1H, CH=N), 7.74 – 7.60 (m, 2H, Ar-H), 7.47 – 7.36 (m, 6H, Ar-H), 7.07 – 6.84 (m, 4H, Ar-H), 5.58 (dd, *J* = 21.0, 9.6 Hz, 1H, CHP), 5.10 (trans), 4.63(cis) (s, 2H, $COCH_2O$), 4.10 – 3.96 (m, 2H, CH_2OP), 3.95 – 3.87 (m, 1H, CHOP), 3.86 – 3.75 (m, 1H, CHOP), 2.41 (s, 3H, CH_3-Hetero), 1.13 (t, *J* = 7.0 Hz, 3H, CH_3), 1.04 (t, *J* = 7.0 Hz, 3H, CH_3). trans:cis=(0.61:0.39); ^{13}C NMR (151 MHz, DMSO) δ 169.03 (s), 164.61 (d, *J* = 9.6 Hz), 164.25 (s), 157.89 (s), 157.43 (s), 150.55 (s), 148.04 (s), 143.86 (s), 134.18 (s), 134.04 (s), 130.37 (s), 130.24 (s), 130.01 (s), 129.56 (d, *J* = 5.3 Hz), 129.44 (d, *J* = 5.3 Hz), 128.88 (d, *J* = 4.6 Hz), 128.72 (s), 128.12 (s), 127.60 (s), 127.21 (s), 127.00 (s), 126.28 (s), 121.28 (s), 118.47 (s), 114.54 (s), 114.35 (s), 66.63 (s), 64.84 (s), 62.66 (d, *J* = 6.9 Hz), 62.46 (d, *J* = 6.5 Hz), 54.48 (s), 53.45 (s), 18.04 (s), 16.34 (d, *J* = 5.2 Hz), 16.16 (d, *J* = 5.3 Hz). IR (thin film, cm^{-1}): 3273.9 (s), 3106.5 (s), 2979.7 (s), 2914.9 (s), 1695.5 (s), 1612.8 (s), 1586.6 (s), 1534.4 (s), 1511.5 (s), 1430.5 (s), 1229.8 (s), 1047.5 (s), 1023.7 (s); HRMS (ESI) m/z for ($C_{28}H_{31}N_4O_5PS$ $[M+H]^+$ cacld. 567.1826, found. 567.1824.

6b: Yield 82%, m.p. 160-161 ^{0}C; ^{1}H NMR (400 MHz, DMSO) δ 11.80 (cis), 11.76 (trans) (s, 1H, CONH), 8.92 (d, *J* = 9.7 Hz, 1H, NH-Ar), 8.73 (cis), 8.38 (trans) (s, 1H, CH=N), 8.00 (cis), 7.95 (trans) (dd, *J* = 7.4, 2.1 Hz, 1H, Ph), 7.55 – 7.34 (m, 6H, Ar-H), 6.96 (m, 4H, Ph), 5.60 (dd, *J* = 20.9, 9.5 Hz, 1H, CH-P), 5.15 (trans), 4.67 (cis) (s, 2H, $COCH_2O$), 4.10 – 3.98 (m, 2H, CH_2OP), 3.97 – 3.89 (m, 1H,CHOP), 3.88 – 3.77 (m, 1H, CHOP), 2.43 (s, 3H, CH_3-Ar), 1.19 – 1.12 (m, 3H,

CH_3), 1.06 (m, 3H, CH_3). trans:cis=(0.62:0.38); ^{13}C NMR (151 MHz, $CDCl_3$) δ 169.21 (s), 164.64 (s), 164.58 (s), 164.51 (s), 157.86 (s), 157.39 (s), 150.55 (s), 143.92 (s), 139.86 (s), 133.32 (s), 133.00 (s), 131.68 (s), 131.46 (s), 131.42 (s), 131.31 (s), 130.37 (s), 129.98 (s), 129.93 (s), 129.59 (s), 129.55 (s), 129.46 (s), 129.42 (s), 128.76 (s), 128.15 (s), 127.70 (s), 127.60 (s), 127.14 (s), 127.02 (s), 126.28 (s), 121.28 (s), 118.47 (s), 114.57 (s), 114.38 (s), 66.63 (s), 64.88 (s), 62.66 (d, *J* = 5.0 Hz), 62.45 (d, *J* = 5.0 Hz), 54.48 (s), 53.45 (s), 18.03 (s), 16.34 (d, *J* = 5.0 Hz), 16.16 (d, *J* = 5.0 Hz). trans:cis = (0.62:0.38). IR (thin film, cm^{-1}): 3245.5 (s), 2986.9 (s), 1697.9 (s), 1592.3 (s), 1538.2 (s), 1435.9 (s), 1249.2 (s), 1050.1 (s), 1025.1(s); HRMS (ESI) m/z for ($C_{28}H_{30}ClN_4O_5PS$ $[M+H]^+$ cacld. 601.1436, found. 601.1438.

6c: Yield 92%, m.p. 172-174 ^0C; ^1H NMR (400 MHz, DMSO) δ 11.69 (trans), 11.68 (cis) (s, 1H, CONH), 8.92 (d, *J* = 9.7 Hz, 1H, NH-Ar), 8.57 (cis), 8.21(trans) (s, 1H, CH=N), 7.93 (trans), 7.88 (cis) (t, *J* = 8.8 Hz, 1H, Ar-H), 7.52 – 7.40 (m, 4H, Ar-H), 7.33 – 7.21 (m, 2H, Ar-H), 7.06 – 6.88 (m, 4H, Ar-H), 5.60 (dd, *J* = 21.0, 9.5 Hz, 1H), 5.13 (trans), 4.66 (cis) (s, 2H, $COCH_2O$), 4.10 – 3.98 (m, 2H, CH_2OP), 3.97 – 3.88 (m, 1H, CHOP), 3.87 – 3.78 (m, 1H, CHOP), 2.43 (s, 3H, CH_3-Ar), 1.21 – 1.11 (m, 3H, CH_3), 1.10 – 1.02 (m, 3H, CH_3), trans:cis =(0.61:0.39); ^{13}C NMR (101 MHz, DMSO) δ 169.52 (s), 165.15 – 164.68 (m), 162.44 (d, *J* = 15.8 Hz), 159.87 (s), 158.27 (s), 157.78 (s), 150.94 (s), 141.24 (d, *J* = 4.7 Hz), 137.09 (d, *J* = 4.5 Hz), 132.42 (dd, *J* = 28.1, 8.5 Hz), 130.76 (s), 129.88 (dd, *J* = 11.8, 5.7 Hz), 129.18 (s), 128.57 (s), 127.99 (s), 126.92 (d, *J* = 10.0 Hz), 126.65 (s), 125.34 (s), 122.06 (dd, *J* = 13.4, 10.0 Hz), 121.65 (s), 118.82 (s), 116.40 (dd, *J* = 20.7, 3.4 Hz), 114.96 (s), 114.76 (s), 67.06 (s), 65.25 (s), 62.94 (dd, *J* = 20.2, 6.8 Hz), 55.18 (s), 53.64 (s), 18.39 (s), 16.61 (dd, *J* = 18.1, 5.4 Hz), trans:cis =(0.61:0.39). IR (thin film, cm^{-1}): 3238.3 (s), 2982.1 (s), 1697.1 (s), 1614.7 (s), 1538.4 (s), 1514.4 (s), 1418.6 (s), 1231.9 (s), 1049.0 (s), 1024.5 (s); HRMS (ESI) m/z for ($C_{28}H_{30}FN_4O_5PS$ $[M+H]^+$ cacld. 585.1731, found. 585.1732.

6d: Yield 87%, m.p. 151-153 ^0C ; ^1H NMR (600 MHz, DMSO) δ 11.90 (trans), 11.84(cis), (s, 1H, CONH), 8.90 (dd, *J* = 9.6, 2.5 Hz, 1H, NH-Ar), 8.70 (trans), 8.34(cis), (s, 1H, CH=N), 8.13 – 7.93 (m, 2H, Ar-H), 7.75 (dt, *J* = 21.2, 7.6 Hz, 1H, Ar-H), 7.62 (dt, *J* = 19.9, 7.8 Hz, 1H, Ar-H), 7.50 – 7.35 (m, 3H, Ar-H), 7.06 – 6.83 (m, 4H, Ar-H), 5.58 (dd, *J* = 21.0, 9.5 Hz, 1H, CH-P), 5.10 (cis), 4.66 (trans), (s, 2H, $COCH_2O$), 4.10 – 3.95 (m, 2H, CH_2OP), 3.95 – 3.87 (m, 1H, CHOP), 3.85 – 3.76 (m, 1H, CHOP), 2.41 (s, 3H, CH_3-Ar), 1.20 – 1.08 (m, 3H, CH_3), 1.08 – 0.98 (m, 3H, CH_3), trans:cis =(0.66:0.34); ^{13}C NMR (151 MHz, DMSO) δ 169.73 (s), 165.29 – 164.79 (m), 158.23 (s),

157.81 (s), 150.95 (s), 148.71 (s), 148.47 (s), 143.72 (s), 139.66 (s), 134.20 (s), 133.93 (s), 131.22 (s), 130.98 (s), 130.78 (s), 129.96 (d, *J* = 4.5 Hz), 129.85 (d, *J* = 6.0 Hz), 129.17 (s), 129.07 (s), 129.02 (s), 128.61 (s), 128.54 (s), 128.51 (s), 128.01 (s), 126.68 (s), 125.11 (s), 124.92 (s), 121.68 (s), 118.86 (s), 114.95 (s), 114.75 (s), 66.98 (s), 65.22 (s), 63.06 (d, *J* = 6.7 Hz), 62.86 (d, *J* = 6.4 Hz), 54.91 (s), 53.88 (s), 18.42 (s), 16.73 (d, *J* = 5.2 Hz), 16.55 (d, *J* = 5.3 Hz), trans:cis =(0.66:0.34); IR (thin film, cm^{-1}): 3262.2 (s), 3040.5 (s), 2984.2 (s), 1683.2 (s), 1615.3 (s), 1533.5 (s), 1340.1 (s), 1223.5 (s), 1047.9 (s), 1021.4 (s); HRMS (ESI) m/z for ($C_{27}H_{21}Cl_2N_2O_3PS$ $[M+H]^+$ cacld. 612.1676, found. 612.1681.

6e: Yield 74%, m.p. 205-207 ^0C ; ^1H NMR (400 MHz, DMSO) δ 11.64 (trans), 11.61 (cis) (s, 1H, CONH), 8.92 (d, *J* = 9.6 Hz, 1H, NH-Ar), 8.29 (cis), 7.97 (trans) (s, 1H, CH=NH), 7.70-7.60 (m, 4H, Ph), 7.45 (dd, *J* = 13.9, 7.5 Hz, 3H, Ar-H), 7.07 – 6.89 (m, 4H, Ph), 5.60 (dd, *J* = 21.0, 9.5 Hz, 1H, CH-P), 5.12 (trans), 4.65 (cis) (s, 2H, $COCH_2O$), 4.11 – 3.98 (m, 2H, CH_2OP), 3.97 – 3.89 (m, 1H, CHOP), 3.87 – 3.78 (m, 1H, CHOP), 2.43 (s, 3H, Ar-CH_3), 1.19 – 1.11 (m, 3H, CH_3), 1.10 – 1.02 (m, 3H,CH_3), trans:cis=(0.61:0.39); ^{13}C NMR (151 MHz, DMSO) δ 169.11 (s), 164.60 (d, *J* = 9.8 Hz), 164.37 (s), 157.86 (s), 157.40 (s), 150.55 (s), 146.79 (s), 142.67 (s), 133.41 (d, *J* = 20.0 Hz), 131.87 (d, *J* = 8.9 Hz), 130.37 (s), 129.56 (d, *J* = 6.3 Hz), 129.43(d, *J* = 6.3 Hz), 129.07 (s), 128.90 (s), 128.73 (s), 128.13 (s), 127.60 (s), 126.28 (s), 123.49 (s), 123.22 (s), 121.27 (s), 118.47 (s), 114.53 (s), 114.37 (s), 66.60 (s), 64.83 (s), 62.66 (d, *J* = 6.9 Hz), 62.46 (d, *J* = 6.6 Hz), 54.47 (s), 53.45 (s), 18.04 (s), 16.34 (d, *J* = 5.2 Hz), 16.16 (d, *J* = 5.3 Hz), trans:cis=(0.61:0.39). IR (thin film, cm^{-1}): 3219.6 (s), 3013.2 (s), 1696.9 (s), 1636.5 (s), 1538.3 (s), 1508.6 (s), 1405.2 (s), 1219.3 (s), 1058.5 (s), 1023.7(s); HRMS (ESI) m/z for ($C_{27}H_{21}Cl_2N_2O_3PS$ $[M+H]^+$ cacld. 645.0931, found. 645.0936.

6f: Yield 91%, m.p. 191-193 ^0C; ^1H NMR (400 MHz, DMSO) δ 11.51 (trans), 11.46 (cis) (s, 1H, CONH), 8.92 (d, *J* = 9.6 Hz, 1H, NH-Ar), 8.27 (cis), 7.96 (trans) (s, 1H, CH=N), 7.57 (d, *J* = 8.1 Hz, 2H,Ar-H), 7.45 (d, *J* = 13.9 Hz, 3H, Ar-H), 7.24 (t, *J* = 6.9 Hz, 2H, Ar-H), 7.05 – 6.90 (m, 4H, Ar-H), 5.60 (dd, *J* = 20.8, 9.6 Hz, 1H), 5.11 (trans), 4.64(cis) (s, 2H, $COCH_2O$)), 4.10 – 3.98 (m, 2H, CH_2OP), 3.97 – 3.88 (m, 1H, CHOP), 3.88 – 3.77 (m, 1H, CHOP), 2.43 (s, 3H,CH_3-Hetero), 2.32 (s, 3H, CH_3-Ar), 1.19 –1.13 (m, 3H, CH_3), 1.10 – 1.03 (m, 3H,CH_3), trans:cis=(0.60:0.40); ^{13}C NMR (151 MHz, DMSO) δ 168.91 (s), 164.60 (d, *J* = 9.7 Hz), 164.12 (s), 157.91 (s), 157.44 (s), 150.55 (s), 148.06 (s), 143.96 (s), 140.09 (s), 139.80 (s), 131.40 (d, *J* = 18.7 Hz), 130.37 (s),

129.48 (s), 128.70 (s), 128.09 (s), 127.60 (s), 127.19 (s), 126.99 (s), 126.28 (s), 121.27 (s), 118.47 (s), 114.53 (s), 114.35 (s), 66.62 (s), 64.83 (s), 62.66 (d, *J* = 6.8 Hz), 62.45 (d, *J* = 6.5 Hz), 54.48 (s), 53.45 (s), 21.09 (s), 18.04 (s), 16.34 (d, *J* = 5.0 Hz), 16.16 (d, *J* = 5.3 Hz). IR (thin film, cm^{-1}): 3235.2 (s), 3032.8 (s), 2978.8 (s), 1684.4 (s), 1613.8 (s), 1531.5 (s), 1412.6 (s), 1226.5 (s), 1053.7 (s), 1027.1(s); HRMS (ESI) m/z for ($C_{29}H_{33}N_4O_5PS$ $[M+H]^+$ cacld. 581.1982, found. 581.1984.

6g: Yield 92%, m.p. 233-235 ^0C ; ^1H NMR (600 MHz, DMSO) δ 11.85 (trans), 11.83 (cis) (s, 1H, CONH), 8.90 (d, *J* = 9.6 Hz, 1H, NH-Ar), 8.40 (cis), 8.07(trans) (s, 1H, CH=N), 8.25 (cis), 8.23 (trans) (d, *J* = 11.4, 2H), 7.96 (trans), 7.93 (cis) (d, *J* = 20.9 Hz, 2H), 7.48 – 7.37 (m, 3H), 7.03 – 6.87 (m, 4H), 5.58 (dd, *J* = 20.9, 9.6 Hz, 1H), 5.16 (trans), 4.68 (cis) (s, 2H), 4.09 – 3.96 (m, 2H), 3.94 – 3.87 (m, 1H), 3.85 – 3.77 (m, 1H), 2.40 (s, 3H), 1.14 (trans), 1.12 (cis) (t, *J* = 7.0 Hz, 3H), 1.05 (trans), 1.02 (cis) (t, *J* = 7.0 Hz, 3H); ^{13}C NMR (151 MHz, DMSO) δ 169.86 (s), 165.18 (s), 165.03 (s), 164.97 (s), 158.22 (s), 157.77 (s), 150.93 (s), 148.41 (s), 148.23 (s), 145.95 (s), 141.90 (s), 140.90 (s), 140.75 (s), 130.76 (s), 129.97 (d, *J* = 6.0 Hz), 129.84 (d, *J* = 6.0 Hz), 129.18 (s), 128.60 (s), 128.56 (s), 128.39 (s), 127.99 (s), 126.68 (s), 124.51 (s), 124.45 (s), 121.68 (s), 118.87 (s), 114.93 (s), 114.79 (s), 66.98 (s), 65.24 (s), 63.08 (s), 62.86 (d, *J* = 6.4 Hz), 54.87 (s), 53.84 (s), 18.43 (s), 16.74 (d, *J* = 5.1 Hz), 16.56 (d, *J* = 5.3 Hz). IR (thin film, cm^{-1}): 3227.8 (s), 2985.1 (s), 2926.6 (s), 1697.7 (s), 1586.1 (s), 1534.6 (s), 1341.6 (s), 1224.6 (s), 1054.9 (s), 1023.5 (s); HRMS (ESI) m/z for ($C_{28}H_{30}N_5O_7PS$ $[M+H]^+$ cacld. 612.1676, found. 612.1679.

6h: Yield 83%, m.p. 173-175 ^0C; ^1H NMR (400 MHz, DMSO) δ 11.55 (trans), 11.51 (cis) (s, 1H, CONH), 8.92 (d, *J* = 9.6 Hz, 1H, NH-Ar), 8.27 (cis), 7.96 (trans) (s, 1H, CH=N), 7.55 – 7.37 (m, 5H, Ar-H), 7.35 – 7.19 (m, 2H, Ar-H), 7.05– 6.88(m, 4H, Ar-H), 5.60 (dd, *J* = 21.0, 9.6 Hz, 1H, CHP), 5.13 (trans), 4.65 (cis) (s, 2H, $COCH_2O$), 4.11 – 3.97 (m, 2H, CH_2OP), 3.96 – 3.87 (m, 1H, CHOP), 3.86 – 3.78 (m, 1H, CHOP), 2.43 (s, 3H, CH_3-Hetero), 2.33 (cis), 2.32 (trans) (s, 3H, CH_3-Ar), 1.19 – 1.12 (m, 3H, CH_3), 1.10 – 1.02 (m, 3H, CH_3), trans:cis = (0.60:0.40); ^{13}C NMR (151 MHz, DMSO) δ 169.40 (s), 165.00 (d, *J* = 9.7 Hz), 164.61 (s), 158.31 (s), 157.83 (s), 150.95 (s), 148.46 (s), 144.43 (s), 138.51 (s), 134.53 (s), 134.37 (s), 131.35 (s), 131.13 (s), 130.77 (s), 129.96 (d, *J* =5.6 Hz), 129.83 (d, *J* =5.6 Hz), 129.17 (d, *J* = 4.8 Hz), 129.13 (d, *J* = 5.6 Hz), 128.50 (s), 128.00 (s), 127.82 (d, *J* = 3.0 Hz), 126.68 (s), 125.06 (s), 124.69 (s), 121.67 (s), 118.86 (s), 114.94 (s), 114.75 (s), 67.03 (s), 65.24 (s), 63.06 (d, *J* = 6.8 Hz), 62.85 (d, *J* = 6.5 Hz), 54.88 (s), 53.85 (s), 21.34 (d, *J* = 1.9 Hz), 18.43 (s), 16.74 (d, *J* = 5.1 Hz), 16.56 (d, *J* = 5.2 Hz). IR (thin

film, cm^{-1}): 3235.2 (s), 3032.8 (s), 2978.8 (s), 1684.4 (s), 1613.8 (s), 1531.5 (s), 1412.6 (s), 1226.5 (s), 1053.7 (s), 1027.1(s); HRMS (ESI) m/z for ($C_{29}H_{33}N_4O_5PS$ $[M+H]^+$ cacld. 581.1982, found. 581.1979.

6i: Yield 87%, m.p. 172-174 ^{0}C; ^{1}H NMR (600 MHz, DMSO) δ 11.66 (s, 1H, CONH), 8.89 (d, *J* = 9.2 Hz, 1H, NH, NH-Ar), 8.27 (s, cis), 7.95 (s, trans) (s, 1H, CH=N), 7.76 (s, trans), 7.70(s, cis) (s, 1H), 7.62 (t, *J* = 5.5 Hz, 1H), 7.48 – 7.40 (m, 5H), 7.03 – 6.87 (m, 4H), 5.58 (dd, *J* = 20.7, 9.3 Hz, 1H), 5.13 (s, trans), 4.64 (s, cis) (s, 2H, Ph-CH_2O), 4.08 – 3.94 (m, 2H), 3.94 – 3.86 (m, 1H), 3.85 – 3.75 (m, 1H), 2.40 (s, 3H), 1.13 (t, *J* = 7.0 Hz, 3H), 1.04 (t, *J* = 7.0 Hz, 3H); ^{13}C NMR (151 MHz, DMSO) δ 169.66 (s), 165.03 (s), 164.96 (s), 164.89 (s), 158.27 (s), 150.94 (s), 150.92(s), 146.73 (s), 142.65 (s), 136.84 (s), 136.69 (s), 134.13 (s), 134.10 (s), 131.20 (s), 131.11 (s), 130.76 (s), 130.26 (s), 130.04 (s), 129.98 (s), 129.84 (s), 129.80 (s), 129.14 (s), 128.50 (s), 128.00 (s), 126.85 (s), 126.67 (s), 126.29(s), 126.22 (s), 121.67 (s), 118.86 (s), 114.93 (s), 114.78 (s), 65.98 (s), 65.26 (s), 63.05 (d, *J* = 7.0 Hz), 62.85 (d, *J* = 7.0 Hz), 54.87 (s), 53.85 (s), 18.43 (s), 16.74 (d, *J* = 5.2 Hz), 16.55 (d, *J* = 5.2 Hz). IR (thin film, cm^{-1}): 3220.0 (s), 3034.8 (s), 2983.4 (s), 1697.0 (s), 1591.3 (s), 1537.9 (s), 1510.7 (s), 1410.6 (s), 1234.5 (s), 1025.1 (s), 1022.9 (s); HRMS (ESI) m/z for ($C_{28}H_{30}ClN_4O_5PS$ $[M+H]^+$ cacld. 601.1436, found. 601.1433.

6j: Yield 87%, m.p. 172-174 ^{0}C; ^{1}H NMR (400 MHz, DMSO) δ 11.81 (s, 1H, CONH), 8.91 (d, *J* = 9.5 Hz, 1H, NH-Ar), 8.50 (s, 1H, Ar-H), 8.44 (cis), 8.12(trans)(s, 1H, N=CH), 8.24 (t, *J* = 8.8 Hz, 1H), 8.17 (trans), 8.11(cis) (d, *J* = 7.9 Hz, 1H, Ar-H), 5.60 (dd, *J* = 21.0, 9.8 Hz, 1H, CHP), 5.19 (trans), 4.69(cis) (s, 2H, $COCH_2O$), 4.12 – 3.98 (m, 2H, CH_2OP), 3.96 – 3.89 (m, 1H, CHOP), 3.86 – 3.78 (m, 1H, CHOP), 2.43 (s, 3H, CH_3-Hetero), 1.15 (t, *J* = 7.0 Hz, 3H, CH_3), 1.06 (t, *J* = 7.0 Hz, 3H, CH_3), trans:cis=(0.60:0.40); ^{13}C NMR (151 MHz, DMSO) δ 169.36 (s), 164.66 (d, *J* = 9.5 Hz), 164.57 (s), 157.86 (s), 157.39 (s), 150.54 (s), 148.30 (d, *J* = 6.4 Hz), 145.63 (s), 141.61 (s), 136.00 (d, *J* = 19.9 Hz), 133.44 (s), 132.93 (s), 130.48 (d, *J* = 16.9 Hz), 130.36 (s), 129.57 (d, *J* = 5.2 Hz), 129.43 (d, *J* = 5.2 Hz), 128.77 (s), 128.15 (s), 127.60 (s), 126.28 (s), 124.43 (s), 124.23 (s), 121.41 (s), 121.28 (s), 121.15 (s), 118.47 (s), 114.53 (s),114.38 (s), 66.58 (s), 64.85 (s), 62.68 (s), 62.46 (d, *J* = 6.4 Hz), 54.48 (s), 53.45 (s), 18.03 (s), 16.34 (d, *J* = 5.2 Hz), 16.16 (d, *J* = 5.4 Hz). IR (thin film, cm^{-1}): 3236.2 (s), 3135.1(s), 2982.8 (s), 1701.2 (s), 1616.5 (s), 1534.1 (s), 1347.5 (s), 1236.9 (s), 1047.9 (s), 1015.4 (s); HRMS (ESI) m/z for ($C_{28}H_{30}N_5O_7PS$ $[M+H]^+$ cacld. 612.1676, found. 612.1679.

6k: Yield 94%, m.p. 182-184 ^{0}C; ^{1}H NMR (400 MHz, DMSO) δ 11.74 (trans), 11.48 (cis) (s, 1H, CONH), 10.76 (trans), 9.78(cis), (s, 1H, OH), 8.96 – 8.88 (m, 1H, NH-Ar), 8.49 (trans), 8.26 (cis) (s, 1H, CH=N), 7.53 – 7.27 (m, 4H, Ar-H), 7.11 – 6.87 (m, 5H, Ar-H), 6.79 (t, *J* = 8.0 Hz, 1H, Ar-H), 5.63 (d, *J* = 9.6 Hz, 1H, CHP), 5.10 (cis), 4.67(trans) (s, 2H, $COCH_2O$), 4.11 – 3.98 (m, 2H, CH_2OP), 3.97 – 3.88 (m, 1H,) CHOP, 3.86 – 3.78 (m, 1H, CHOP), 2.43 (s, 3H,CH_3-Hetero), 2.22(cis),2.20(trans) (s, 3H, CH_3-Ar), 1.15 (t, *J* = 7.0 Hz, 3H), 1.06(cis), 1.07(trans) (t, *J* = 7.0 Hz, 3H). trans:cis=(0.60:0.40); ^{13}C NMR (101 MHz, DMSO) δ 169.51 (s), 165.14 – 164.58 (m), 150.93 (s), 141.25 (s), 137.09 (s), 132.42 (dd, *J* = 27.7, 8.4 Hz), 130.76 (s), 129.88 (dd, *J* = 11.7, 5.7 Hz), 128.56 (s), 127.99 (s), 126.92 (d, *J* = 8.1 Hz), 126.65 (s), 125.34 (s), 121.99 (d, *J* = 10.2 Hz), 121.65 (s), 118.83 (s), 116.51 (d, *J* = 3.2 Hz), 114.86 (d, *J* = 20.7 Hz), 67.04 (s), 65.24 (s), 62.94 (dd, *J* = 20.3, 6.9 Hz), 55.16 (s), 18.39 (s), 16.62 (dd, *J* = 18.1, 5.4 Hz). IR (thin film, cm^{-1}): 3235.7 (s), 2977.1 (s), 2912.6 (s), 1698.9 (s), 1617.1 (s), 1539.4 (s), 1222.1 (s), 1052.8 (s), 1021.4 (s); HRMS (ESI) m/z for ($C_{29}H_{33}N_4O_6PS$ $[M+H]^+$ cacld. 597.1931, found. 597.1936.

6l: Yield 77%, m.p. 172-173 ^{0}C; ^{1}H NMR (600 MHz, DMSO) δ 11.45 (s, trans), 11.41 (s, cis) (s, 1H, CONH), 10.36 (s, cis), 10.31(s, trans) (s, 1H, OH), 8.93 (d, *J* = 9.6 Hz, 1H, NH-Ar), 8.17 (s, cis), 7.85 (s, trans) (s, 1H, CH=N), 7.58 – 7.20 (m, 5H), 7.04 – 6.85 (m, 5H), 5.58 (dd, *J* = 20.9, 9.4 Hz, 1H), 5.08 (s, trans), 4.60 (s, cis) (s, 2H, Ph-CH_2O), 4.08 – 3.95 (m, 2H), 3.94 – 3.86 (m, 1H), 3.85 – 3.75 (m, 1H), 2.41 (s, trans), 2.40 (s, cis) (s, 3H, CH_3), 1.13 (t, trans), 1.12 (t, cis) (t, *J* = 7.0 Hz, 3H, CH_3), 1.04 (t, trans), 1.03 (t, cis) (t, *J* = 7.0 Hz, 3H, CH_3); ^{13}C NMR (151 MHz, DMSO) δ 169.25 (s), 165.03 (s), 164.42 (s), 158.30 (s), 157.84 (s), 152.45 (s), 152.37 (s), 150.94 (s), 150.85 (s), 150.77 (s), 147.60 (s), 147.52 (s), 147.44 (s), 147.29 (s), 147.21 (s), 143.38 (s), 130.76 (s), 129.95 (d, *J* = 4.8Hz), 129.82 (d, *J* = 4.8Hz), 129.09 (s), 128.45 (s), 127.99 (s), 126.68 (s), 126.34 (s), 126.30 (s), 124.88 (s),124.84 (s), 121.69 (s), 121.67 (s), 118.86 (s), 118.35 (s), 118.27 (s), 114.92 (s), 114.74 (s), 114.65 (s), 114.52 (s), 114.38 (s), 114.25 (s), 67.01 (s), 65.18 (s), 63.05 (d, *J* = 7.0 Hz), 62.85 (d, *J* = 6.8 Hz), 54.87 (s), 53.85 (s), 18.43 (s), 16.73 (d, *J* = 5.2 Hz), 16.55 (d, *J* = 5.2 Hz); IR (thin film, cm^{-1}): 3235.6 (s), 2983.7(s), 1690.9 (s), 1616.8 (s), 1531.8 (s), 1301.1 (s), 1248.5 (s), 1035.1 (s), 1027.0 (s); HRMS (ESI) m/z for ($C_{28}H_{30}FN_4O_6PS$ $[M+H]^+$ 601.1681, found. 601.1676.

6m: Yield 84%, m.p. 214-216 ^{0}C; ^{1}H NMR (400 MHz, DMSO) δ 11.48 (s, trans), 11.46 (s, cis) (s, 1H, CONH), 10.71 (s, cis), 10.66 (s, trans) (s, 1H, OH), 8.92 (d, *J* = 9.6 Hz, 1H, NH-Ar), 8.18 (s,

cis), 7.87 (s, trans) (s, 1H, CH=N), 7.70 (d, *J* =1.9 Hz, trans), 7.65 (d, *J* =1.9 Hz, cis), 7.52 – 7.38 (m, 4H, Ar-H), 7.06 – 6.88 (m, 5H,Ar-H), 5.60 (dd, *J* = 20.9, 9.5 Hz, 1H, CHP), 5.11(s, trans), 4.63(s, cis) (s, 2H, Ph-CH_2O), 4.14 – 3.75 (m, 4H, 2CH_2OP), 2.43 (s, 3H, CH_3), 1.15 (td, *J* = 7.0, 1.7 Hz, 3H, CH_3), 1.06 (td, *J* = 7.0, 4.8 Hz, 3H, CH_3);^{13}C NMR (101 MHz, DMSO) δ 169.23 (s, trans), 165.04 (s, cis), 164.94 (s, cis), 164.43 (s, trans), 158.30 (s, trans), 157.83 (s, cis), 155.34 (s, cis), 155.12 (s, trans), 150.93 (s), 147.27 (s), 143.17 (s), 130.75 (s), 129.83 (s), 129.96 (s, cis), 129.90 (s, trans), 129.83 (s, trans), 129.77 (s, cis), 129.01 (s, cis), 128.91(s, trans), 128.62 (s, trans),128.47 (s, cis), 127.99 (s), 127.68 (s, cis), 127.60 (s, trans), 126.89 (s, cis), 126.81 (s, trans), 126.66 (s), 121.67 (s, cis), 121.65 (s, trans), 120.78 (s, trans), 120.73 (s, cis), 118.82 (s), 117.32 (s, cis), 117.25 (s, trans), 114.92 (s, cis), 114.76 (s, trans), 67.05 (s, 40%), 65.22 (s, 60%), 62.94 (dd, *J* = 19.6, 6.8 Hz), 55.41 (d, *J* = 155.54 Hz), 18.39 (s), 16.62 (dd, *J* = 17.8, 5.3 Hz). IR (thin film, cm^{-1}): 3219.3 (s), 2985.9 (s), 1682.3 (s), 1601.7 (s), 1532.8 (s), 1514.0 (s), 1298.6 (s), 1243.4 (s), 1052.0 (s), 1024.8 (s). HRMS (ESI) m/z for ($C_{28}H_{30}ClN_4O_6PS[M+H]^+$ cacld. 617.1385, found. 617.1381.

3、^{1}H and ^{13}C NMR Spectra of 6a-6m

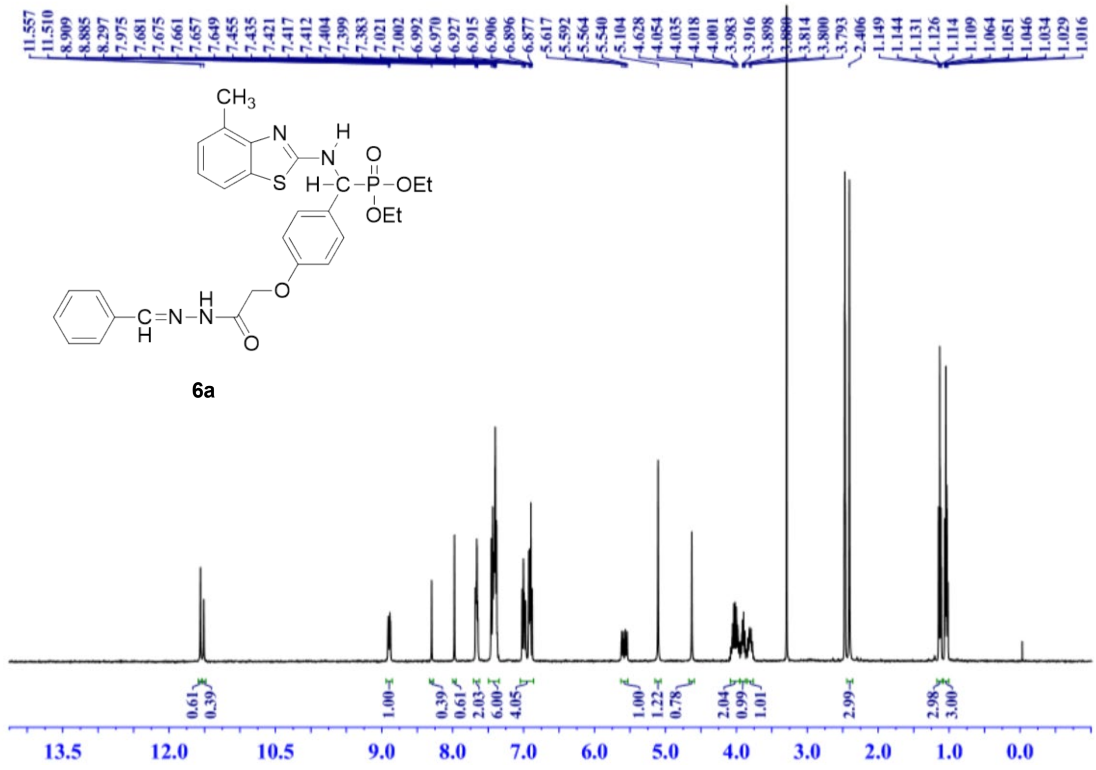

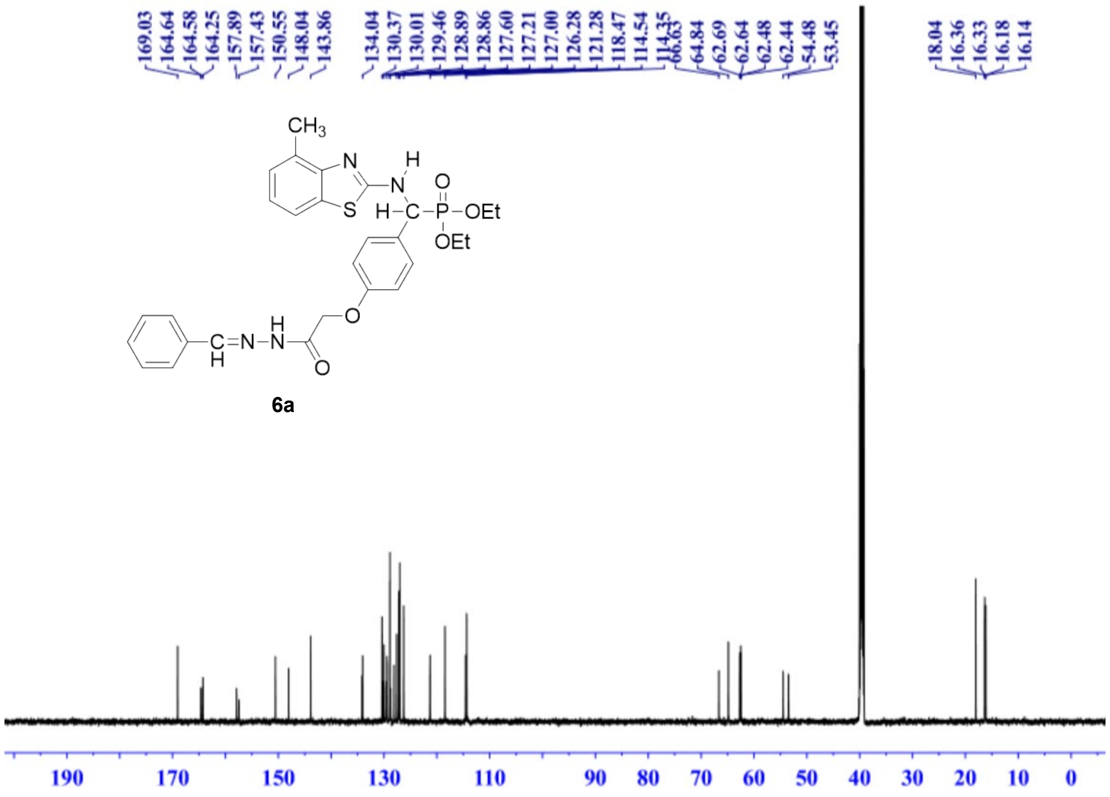

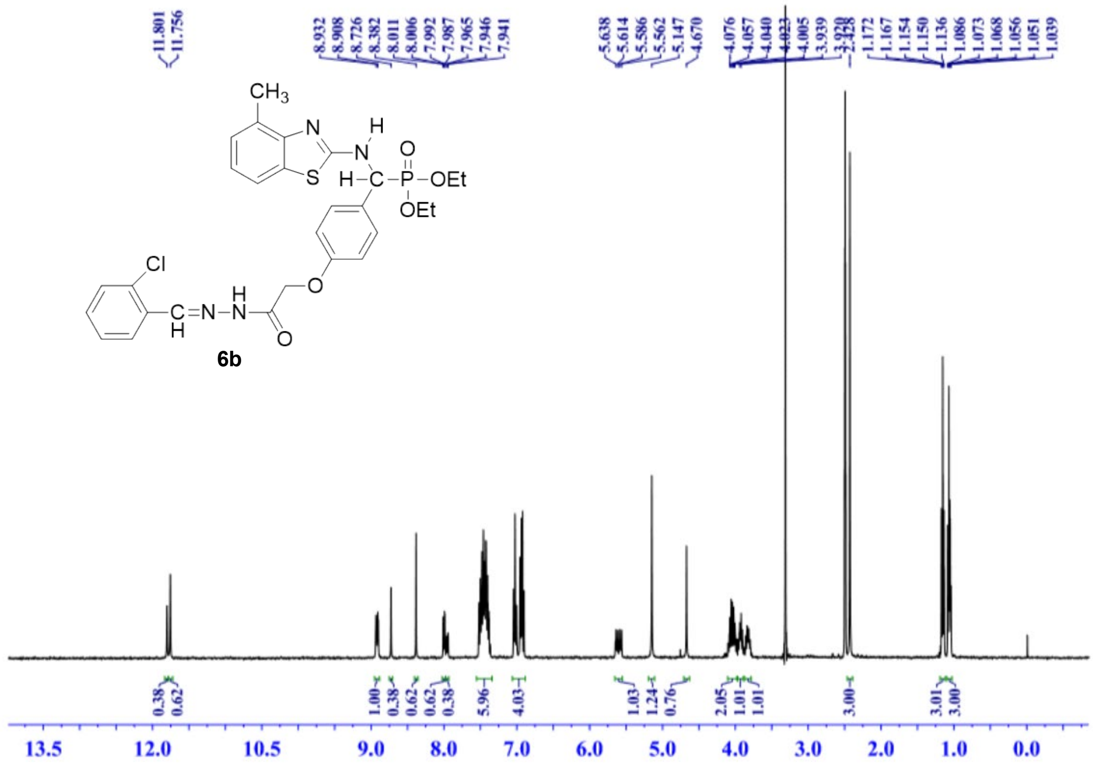
6b

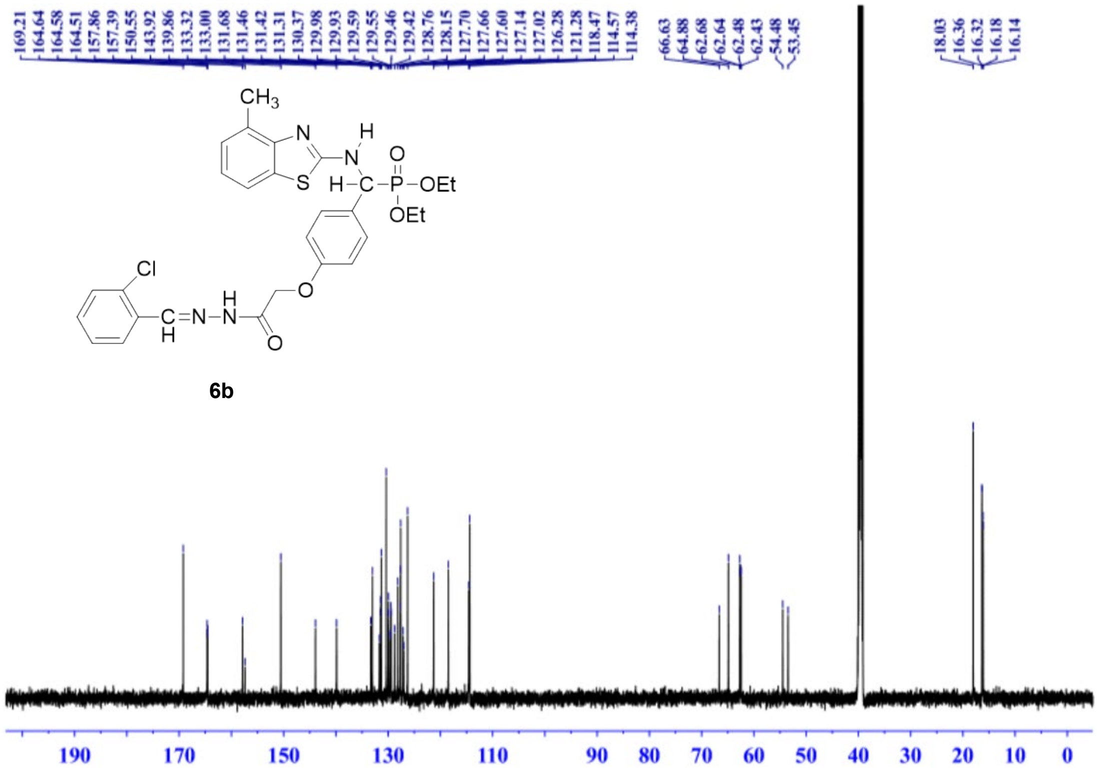
6b

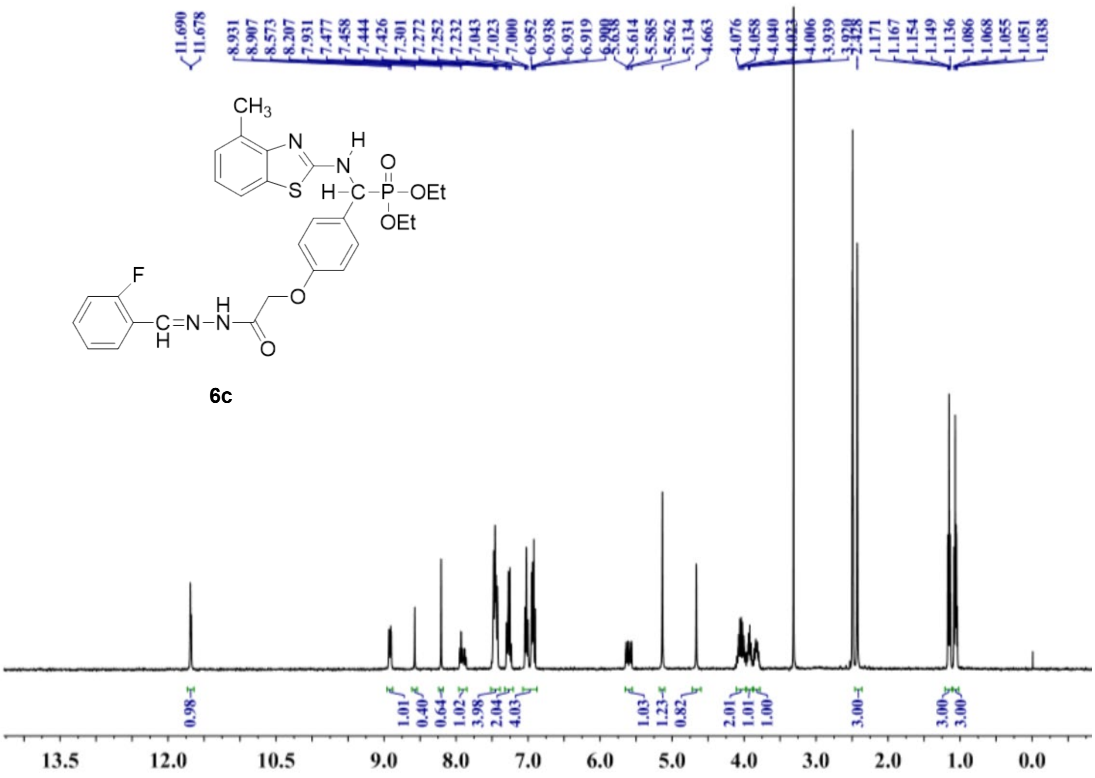
CH3
N
H
O
S
H-C-P-OEt
OEt
F
H
C=N-N
H
O
O
6c
0.98
1.01
0.40
0.64
1.02
3.98
2.04
4.03
1.03
1.23
0.82
2.01
1.01
1.00
3.00
3.00
3.00
13.5
12.0
10.5
9.0
8.0
7.0
6.0
5.0
4.0
3.0
2.0
1.0
0.0

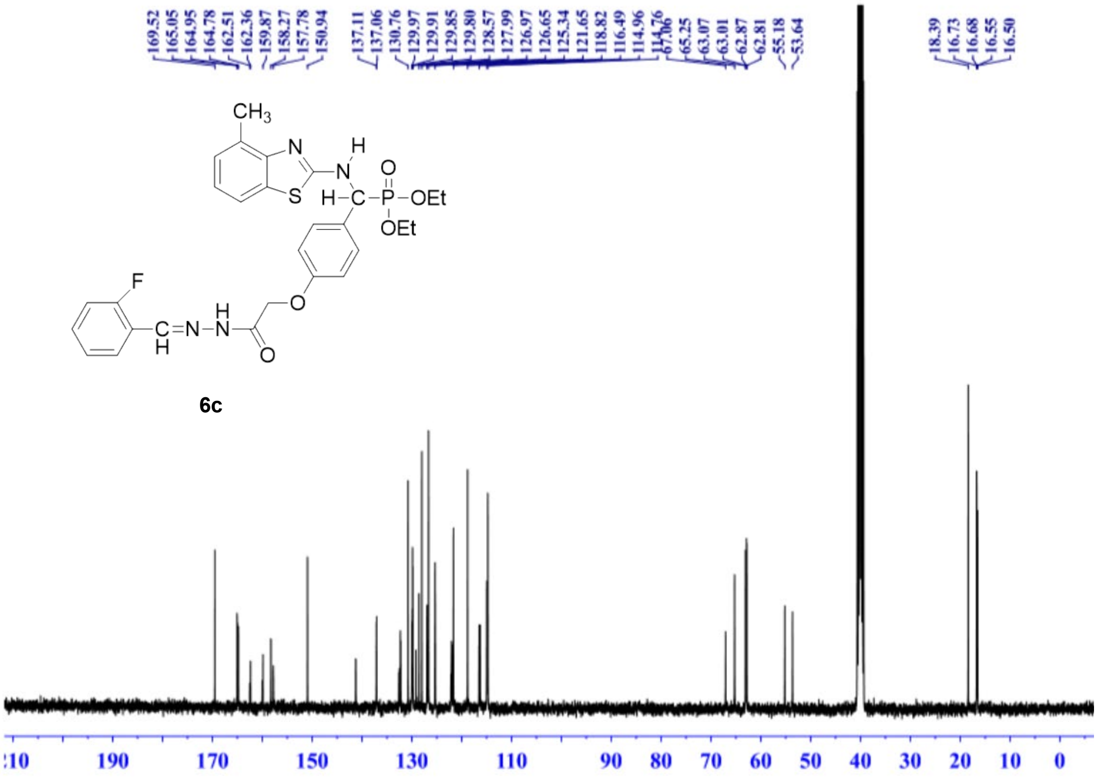
169.52
165.05
164.95
164.78
162.51
162.36
159.87
158.27
157.78
150.94
137.11
137.06
130.76
129.97
129.91
129.85
129.80
128.57
127.99
126.97
126.65
125.34
121.65
118.82
116.49
114.96
65.25
63.07
63.01
62.87
62.81
55.18
53.64
18.39
16.73
16.68
16.55
16.50
CH3
N
H
O
S
H-C-P-OEt
OEt
F
H
C=N-N
H
O
O
6c
190
170
150
130
110
90
80
70
60
50
40
30
20
10
0

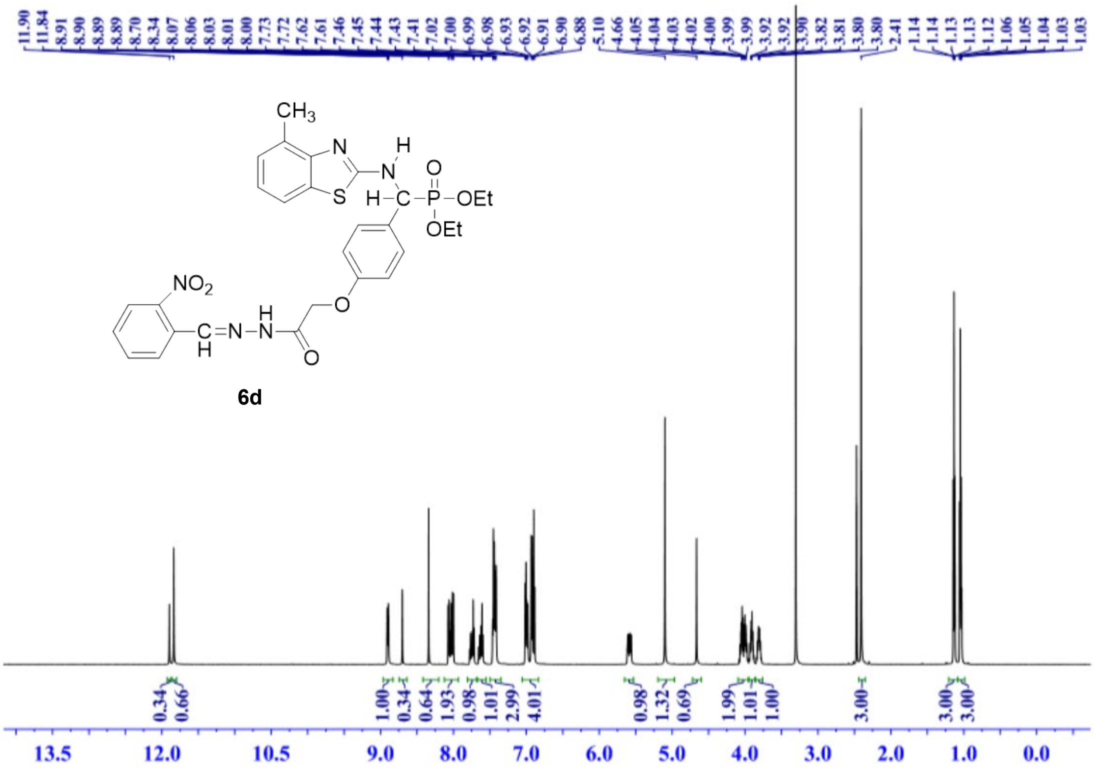
11.90 11.84 8.91 8.90 8.89 8.89 8.70 8.34 8.07 8.06 8.03 8.01 8.00 7.73 7.72 7.62 7.61 7.46 7.45 7.44 7.43 7.41 7.02 7.00 6.99 6.98 6.93 6.92 6.91 6.90 6.88 5.10 4.66 4.05 4.04 4.03 4.02 4.00 3.99 3.99 3.92 3.92 3.90 3.82 3.81 3.80 3.80 2.41 1.14 1.14 1.13 1.13 1.12 1.06 1.05 1.04 1.03 1.03
CH3
N
S
H
O
P
OEt
OEt
NO2
6d
0.34 0.66 1.00 0.34 0.64 1.93 0.98 1.01 2.99 4.01 0.98 1.32 0.69 1.99 1.01 1.00 3.00 3.00 3.00
13.5 12.0 10.5 9.0 8.0 7.0 6.0 5.0 4.0 3.0 2.0 1.0 0.0

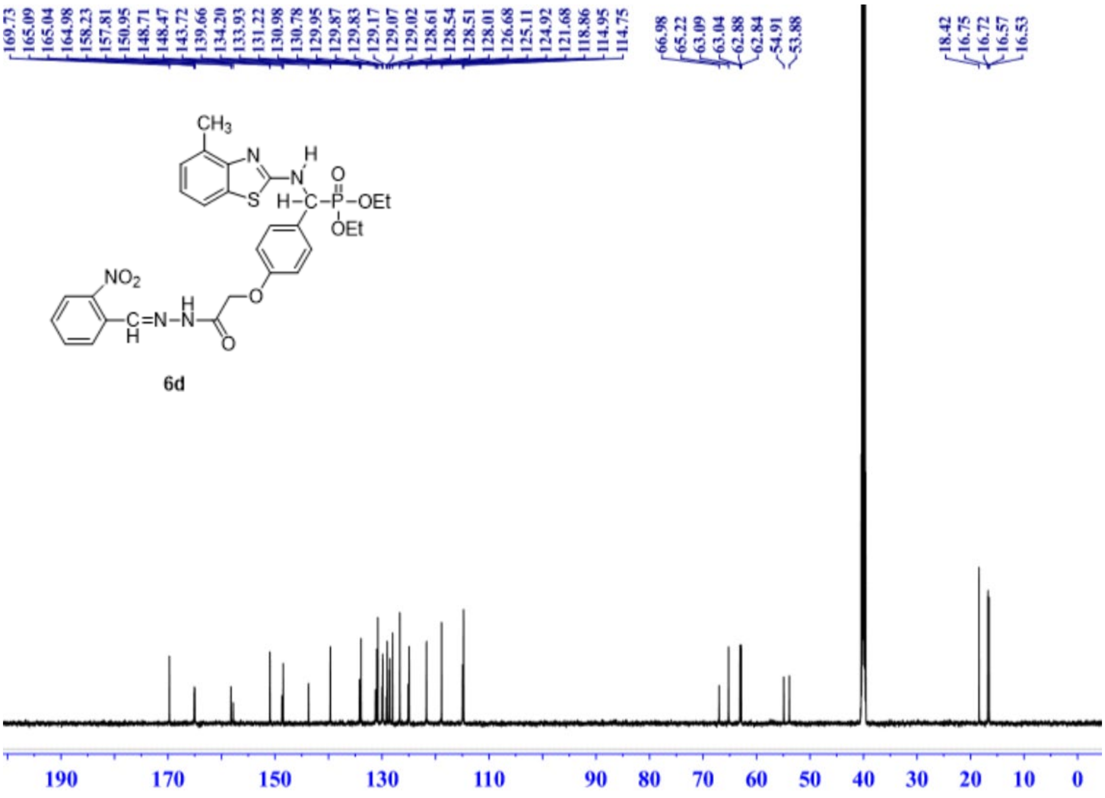
169.73 165.09 165.04 164.98 158.23 157.81 150.95 148.71 148.47 143.72 139.66 134.20 133.93 131.22 130.98 130.78 129.95 129.87 129.83 129.17 129.07 129.02 128.61 128.54 128.51 128.01 126.68 125.11 124.92 121.68 118.86 114.95 114.75 66.98 65.22 63.09 63.04 62.88 62.84 54.91 53.88 18.42 16.75 16.72 16.57 16.53
CH3
NO2
OEt
OEt
6d
190 170 150 130 110 90 80 70 60 50 40 30 20 10 0

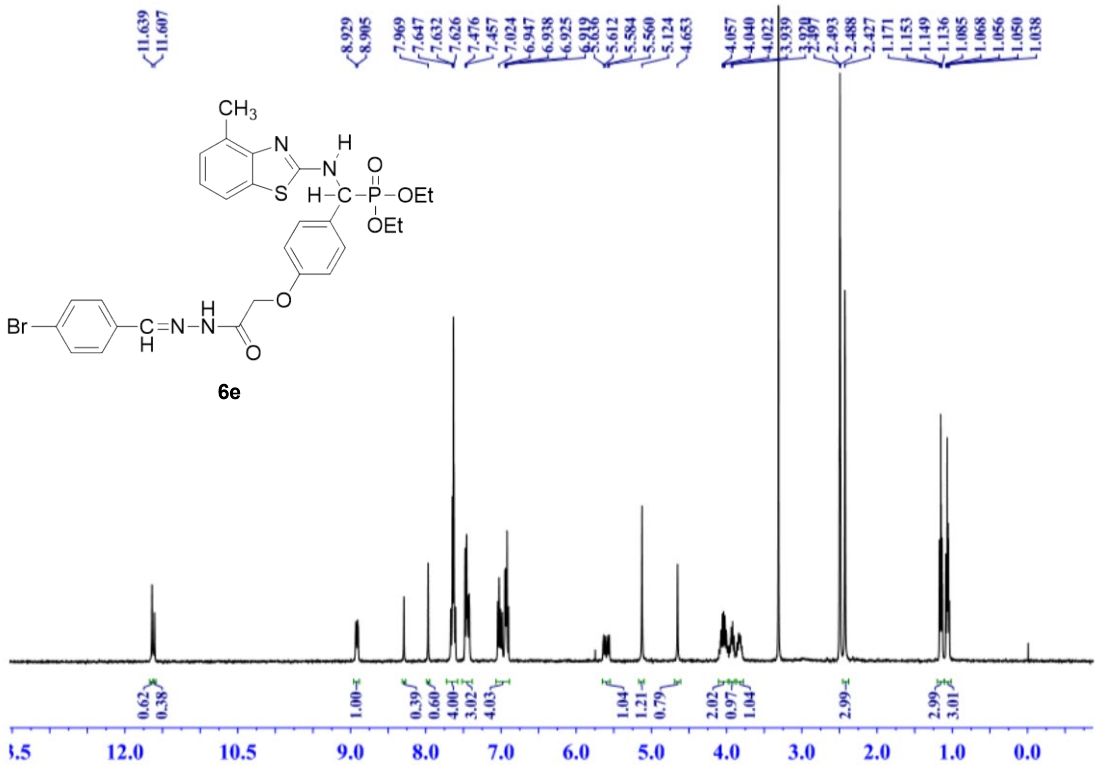
11.639
11.607
8.929
8.905
7.969
7.647
7.632
7.626
7.476
7.457
7.024
6.947
6.938
6.925
5.612
5.584
5.560
5.124
4.653
4.057
4.040
4.022
2.493
2.488
2.427
1.171
1.153
1.149
1.136
1.085
1.068
1.056
1.050
1.038
CH3
N
H
O
S
H-C-P-OEt
OEt
Br
C=N-N
H
6e
0.62
0.38
1.00
0.39
0.60
4.00
3.02
4.03
1.04
1.21
0.79
2.02
0.97
1.04
2.99
2.99
3.01
12.0
10.5
9.0
8.0
7.0
6.0
5.0
4.0
3.0
2.0
1.0
0.0

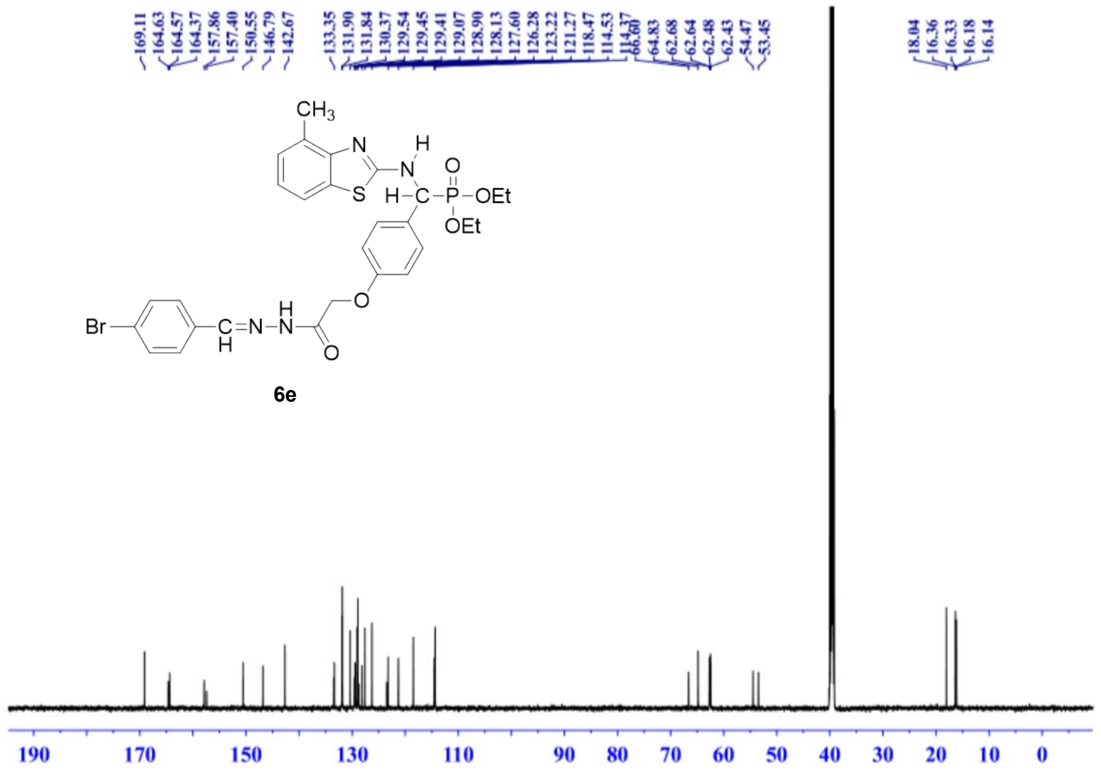
169.11
164.63
164.57
164.37
157.86
157.40
150.55
146.79
142.67
133.35
131.90
131.84
130.37
129.54
129.45
129.41
129.07
128.90
128.13
127.60
126.28
123.22
121.27
118.47
114.53
64.83
62.68
62.64
62.48
62.43
54.47
53.45
18.04
16.36
16.33
16.18
16.14
CH3
N
H
O
S
H-C-P-OEt
OEt
Br
C=N-N
H
6e
190
170
150
130
110
90
80
70
60
50
40
30
20
10
0

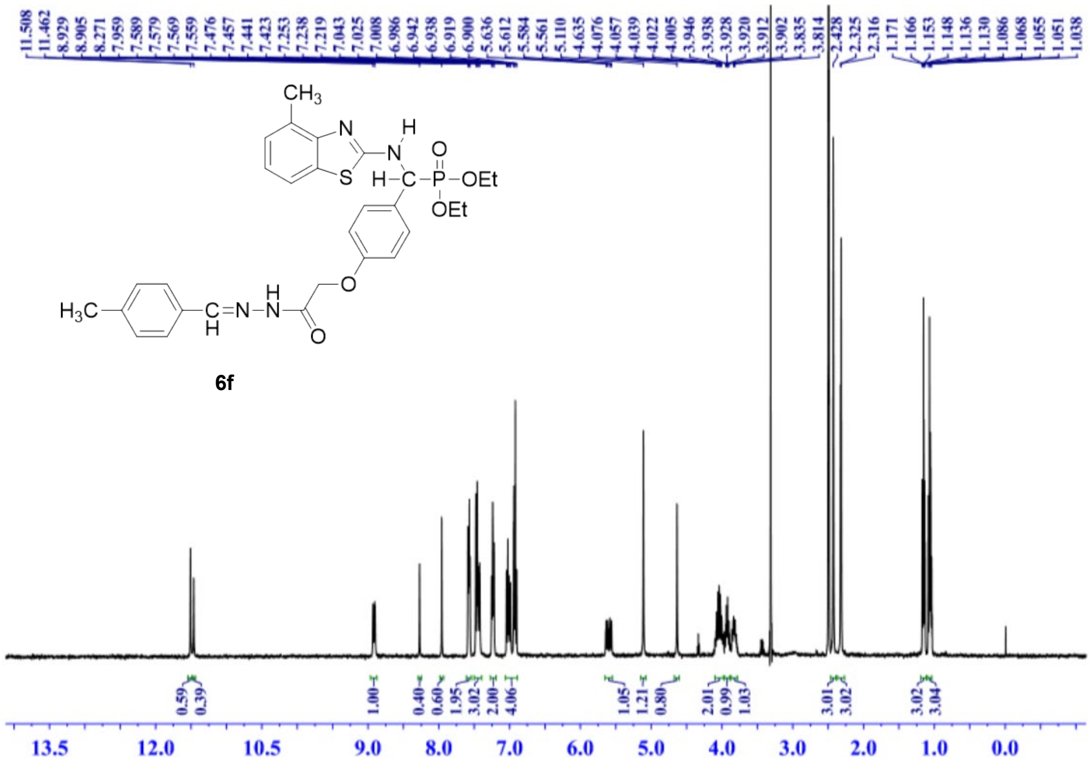
11.508 11.462 8.929 8.905 8.271 7.959 7.589 7.579 7.569 7.559 7.476 7.457 7.441 7.423 7.253 7.238 7.219 7.043 7.025 7.008 6.986 6.942 6.938 6.919 6.900 5.636 5.612 5.584 5.561 5.110 4.635 4.076 4.057 4.039 4.022 4.005 3.946 3.938 3.928 3.920 3.912 3.902 3.835 3.814 2.428 2.325 2.316 1.171 1.166 1.153 1.148 1.136 1.130 1.086 1.068 1.055 1.051 1.038
CH_3
N
H
N
O
S
H–C–P–OEt
OEt
O
H_3C
C=N–N
H
H
O
6f
0.59 0.39 1.00 0.40 0.60 1.95 3.02 2.00 4.06 1.05 1.21 0.80 2.01 0.99 1.03 3.01 3.02 3.02 3.04
13.5 12.0 10.5 9.0 8.0 7.0 6.0 5.0 4.0 3.0 2.0 1.0 0.0

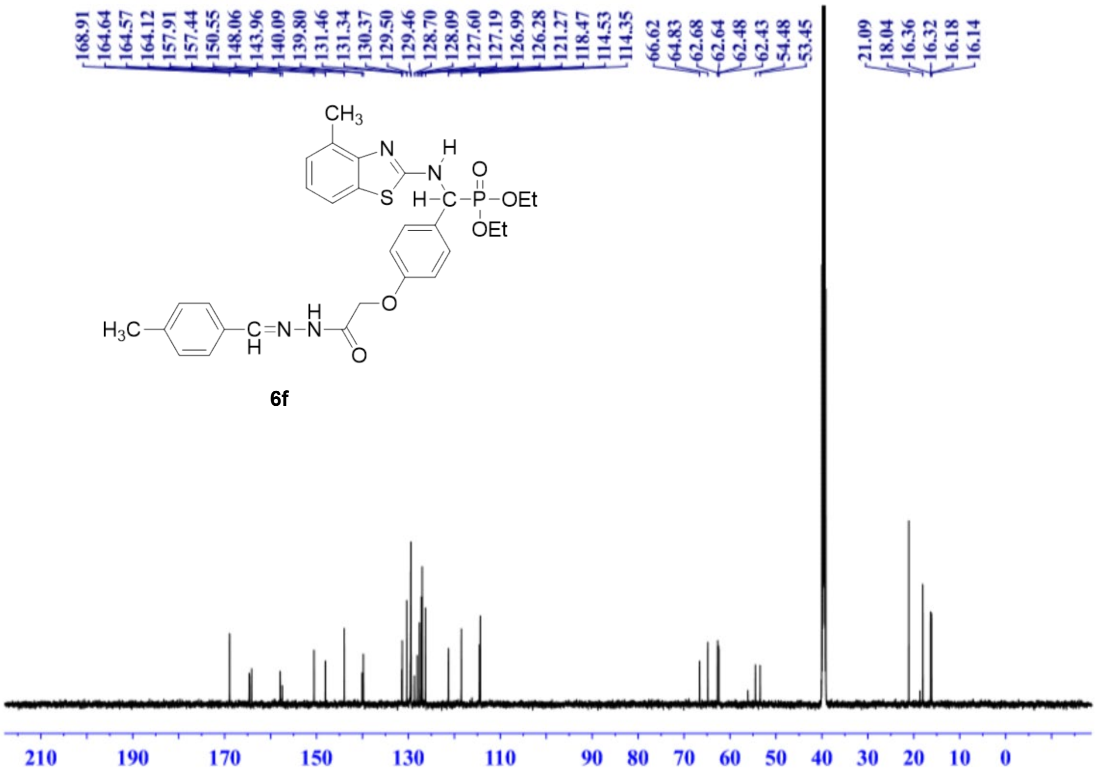
168.91 164.64 164.57 164.12 157.91 157.44 150.55 148.06 143.96 140.09 139.80 131.46 131.34 130.37 129.50 129.46 128.70 128.09 127.60 127.19 126.99 126.28 121.27 118.47 114.53 114.35 66.62 64.83 62.68 62.64 62.48 62.43 54.48 53.45 21.09 18.04 16.36 16.32 16.18 16.14
CH_3
N
H
N
O
S
H–C–P–OEt
OEt
O
H_3C
C=N–N
H
H
O
6f
210 190 170 150 130 110 90 80 70 60 50 40 30 20 10 0

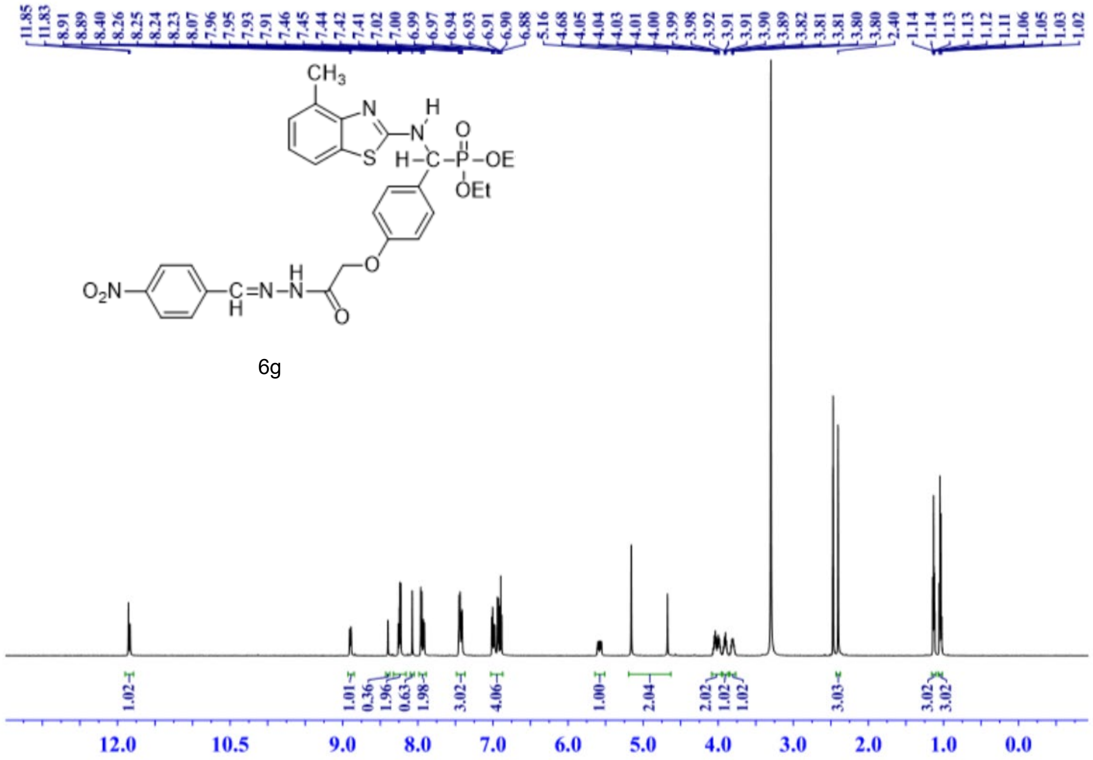
6g
12.0 10.5 9.0 8.0 7.0 6.0 5.0 4.0 3.0 2.0 1.0 0.0

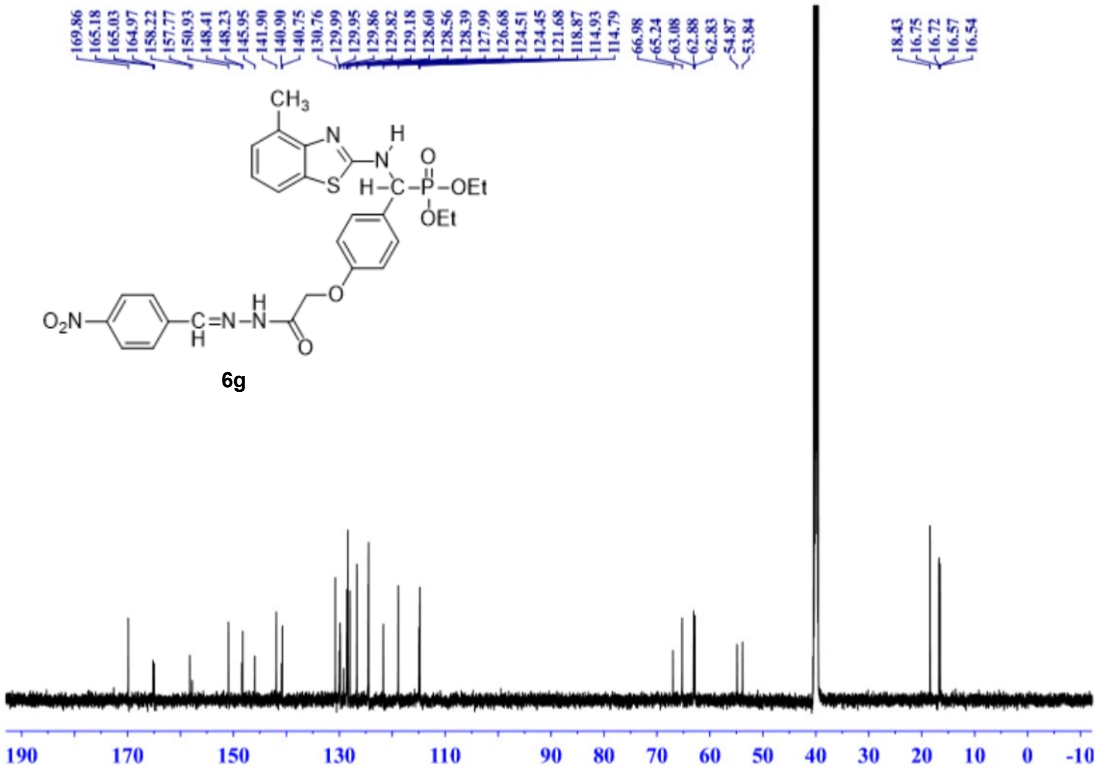
6g
190 170 150 130 110 90 80 70 60 50 40 30 20 10 0 -10

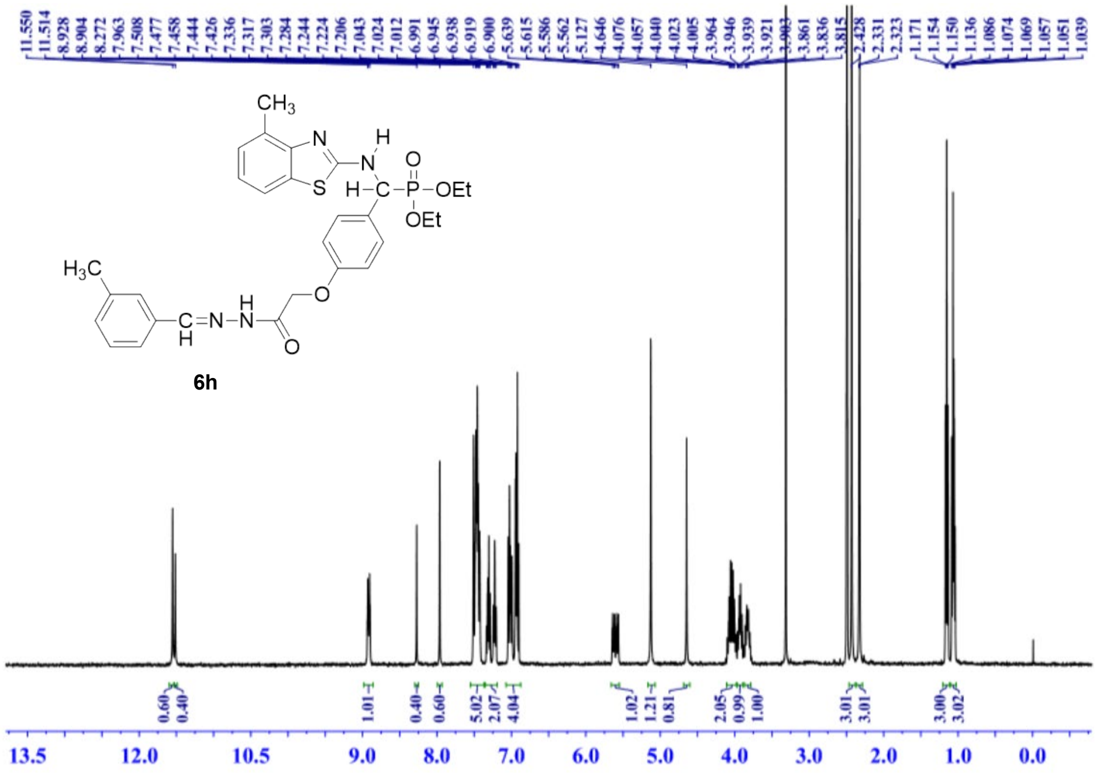
11.550
11.514
8.928
8.904
8.272
7.963
7.508
7.477
7.458
7.444
7.426
7.336
7.317
7.303
7.284
7.244
7.224
7.206
7.043
7.024
7.012
6.991
6.945
6.938
6.919
6.900
5.639
5.615
5.586
5.562
5.127
4.646
4.076
4.057
4.040
4.023
4.005
3.964
3.946
3.939
3.921
3.903
3.861
3.836
3.815
2.428
2.331
2.323
1.171
1.154
1.150
1.136
1.086
1.074
1.069
1.057
1.051
1.039
CH3
N
H
N
S
H-C-P-OEt
O
OEt
H3C
C=N-N
H
H
O
O
6h
0.60
0.40
1.01
0.40
0.60
5.02
2.07
4.04
1.02
1.21
0.81
2.05
0.99
1.00
3.01
3.01
3.00
3.02
13.5
12.0
10.5
9.0
8.0
7.0
6.0
5.0
4.0
3.0
2.0
1.0
0.0

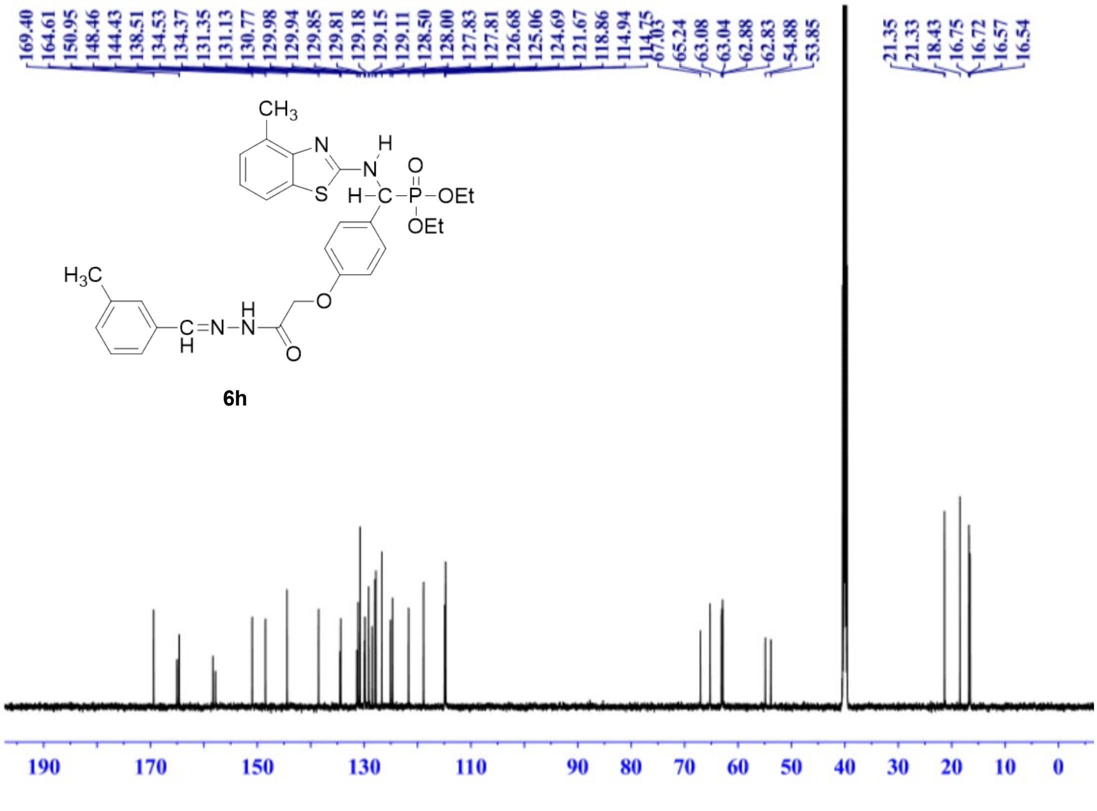
169.40
164.61
150.95
148.46
144.43
138.51
134.53
134.37
131.35
131.13
130.77
129.98
129.94
129.85
129.81
129.18
129.15
129.11
128.50
128.00
127.83
127.81
126.68
125.06
124.69
121.67
118.86
114.94
114.75
67.03
65.24
63.08
63.04
62.88
62.83
54.88
53.85
21.35
21.33
18.43
16.75
16.72
16.57
16.54
CH3
N
H
N
S
H-C-P-OEt
O
OEt
H3C
C=N-N
H
H
O
O
6h
190
170
150
130
110
90
80
70
60
50
40
30
20
10
0

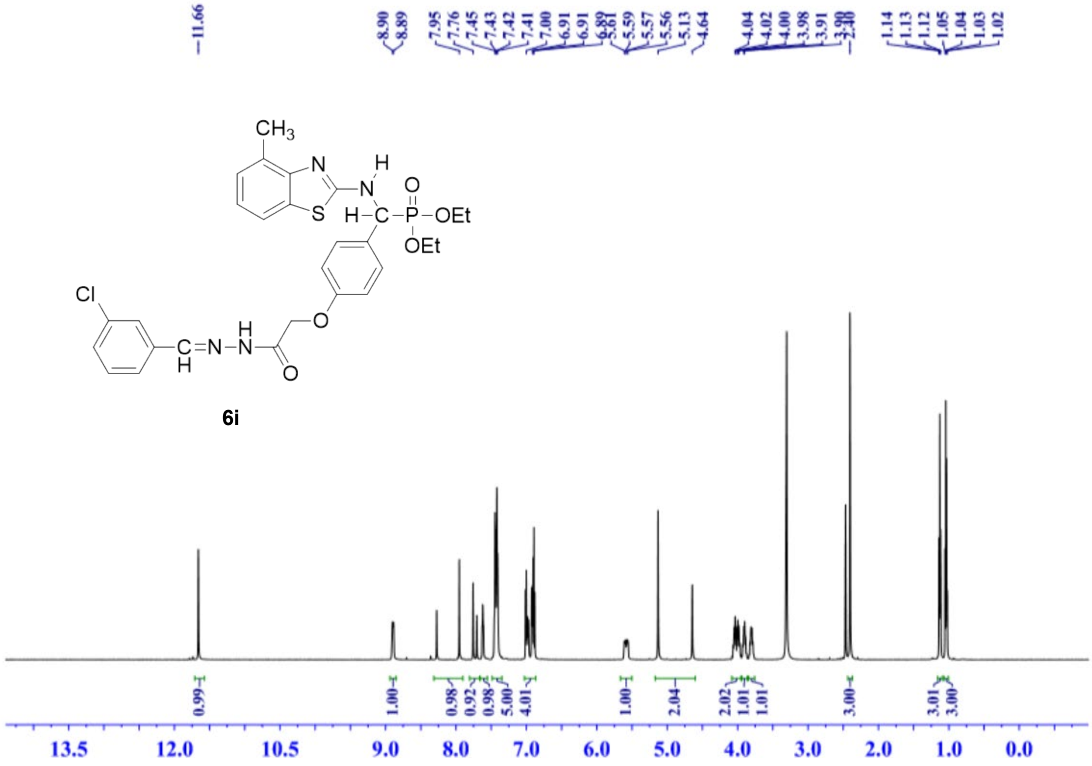
CH3
N
H
N
O
P
OEt
S
H
C
OEt
Cl
O
H
C=N-N
H
O
6i

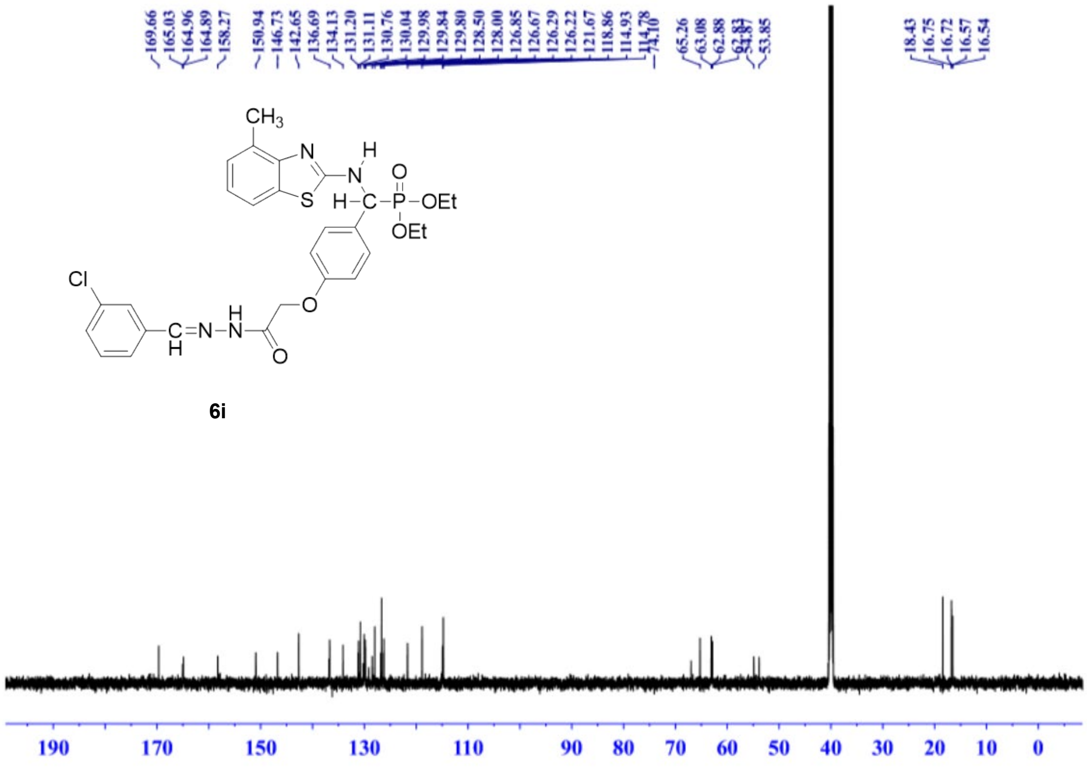
CH3
N
H
N
O
P
OEt
S
H
C
OEt
Cl
O
H
C=N-N
H
O
6i

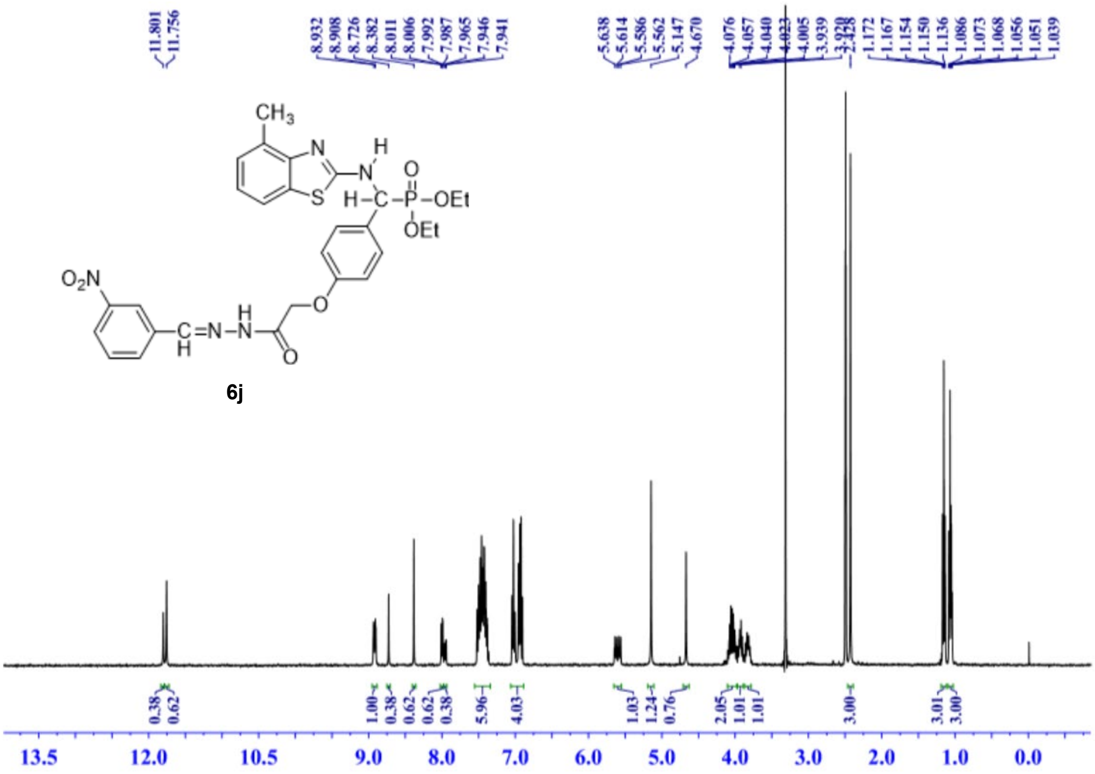
CH3
N
S
H
N
H-C-P-OEt
O
OEt
O2N
C=N-N
H
O
6j

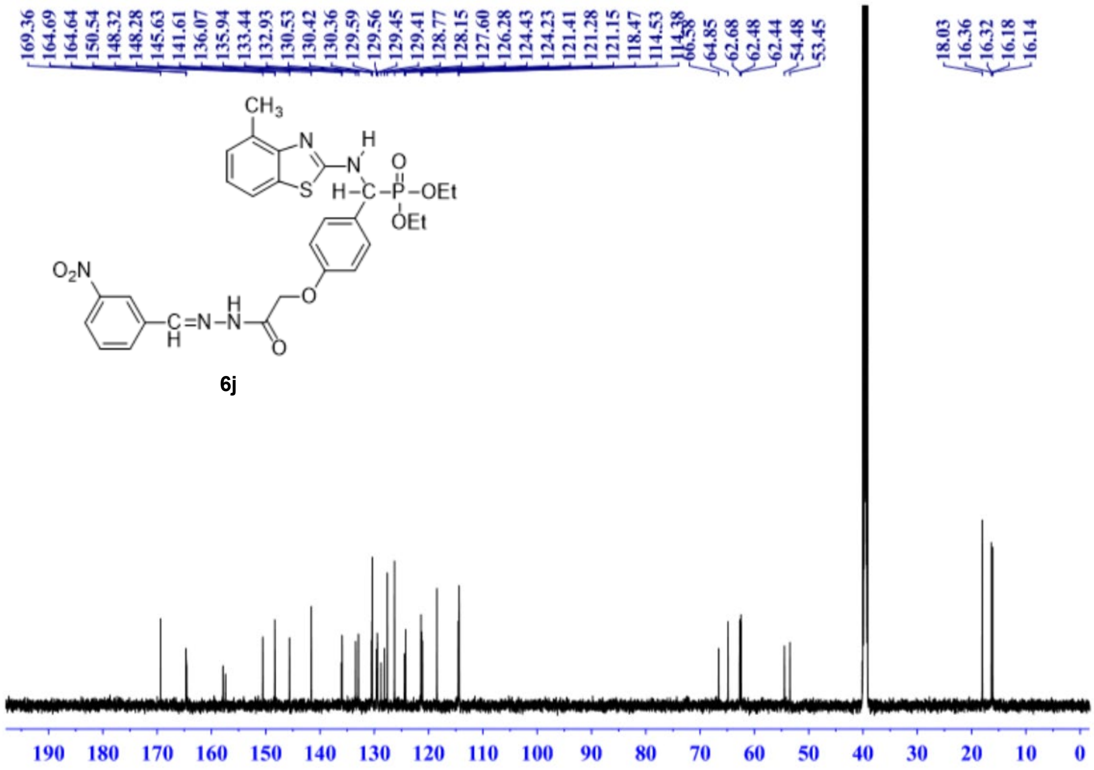
CH3
N
S
H
N
H-C-P-OEt
O
OEt
O2N
C=N-N
H
O
6j

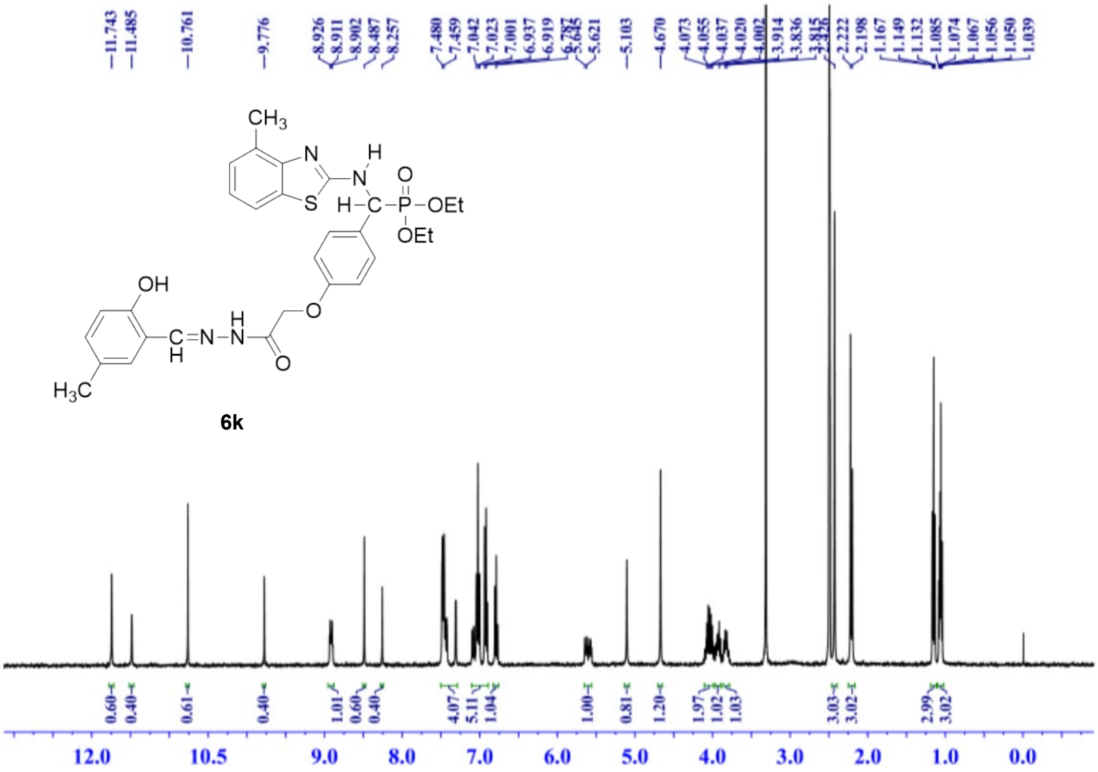
11.743
11.485
10.761
9.776
8.926
8.911
8.902
8.487
8.257
7.480
7.459
7.042
7.023
7.001
6.937
6.919
5.621
5.103
4.670
4.073
4.055
4.037
4.020
3.914
3.836
2.222
2.198
1.167
1.149
1.132
1.085
1.074
1.067
1.056
1.050
1.039
CH3
N
H
N
O
S
H-C-P-OEt
OEt
OH
H
C=N-N
H
O
O
H3C
6k
0.60
0.40
0.61
0.40
1.01
0.60
0.40
4.07
5.11
1.04
1.00
0.81
1.20
1.97
1.02
1.03
3.03
3.02
2.99
3.02
12.0
10.5
9.0
8.0
7.0
6.0
5.0
4.0
3.0
2.0
1.0
0.0

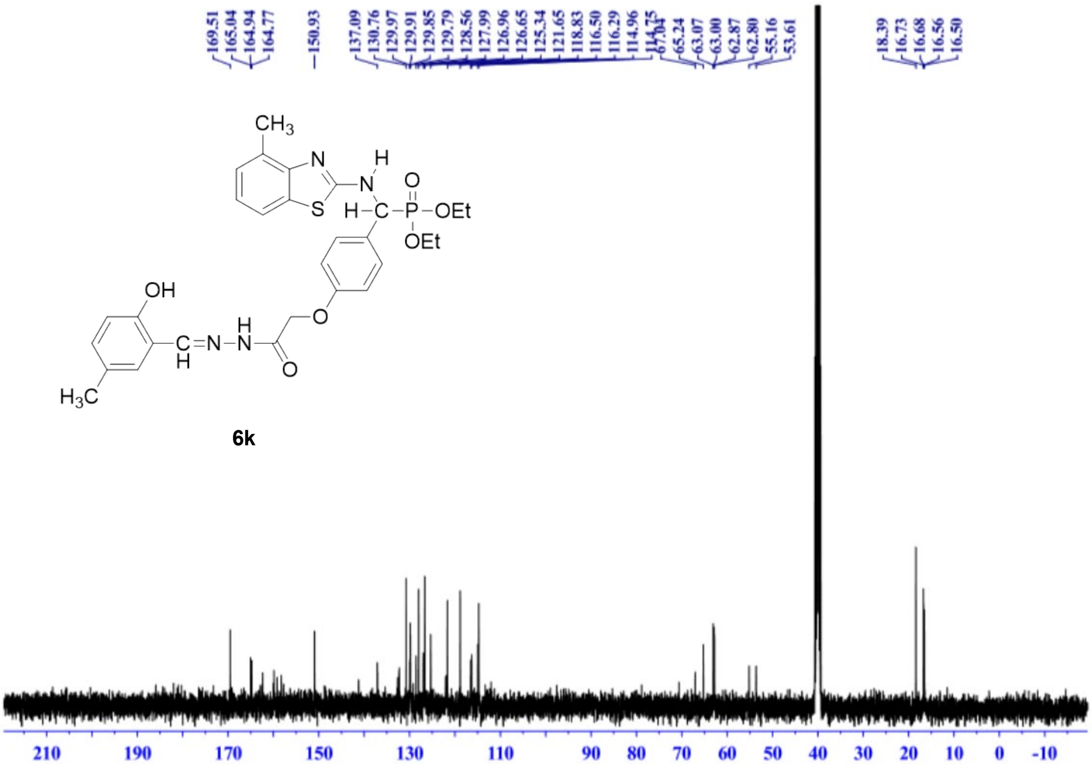
169.51
165.04
164.94
164.77
150.93
137.09
130.76
129.97
129.91
129.85
129.79
128.56
127.99
126.96
126.65
125.34
121.65
118.83
116.50
116.29
114.96
65.24
63.07
63.00
62.87
62.80
55.16
53.61
18.39
16.73
16.68
16.56
16.50
CH3
N
H
N
O
S
H-C-P-OEt
OEt
OH
H
C=N-N
H
O
O
H3C
6k
210
190
170
150
130
110
90
80
70
60
50
40
30
20
10
0
-10

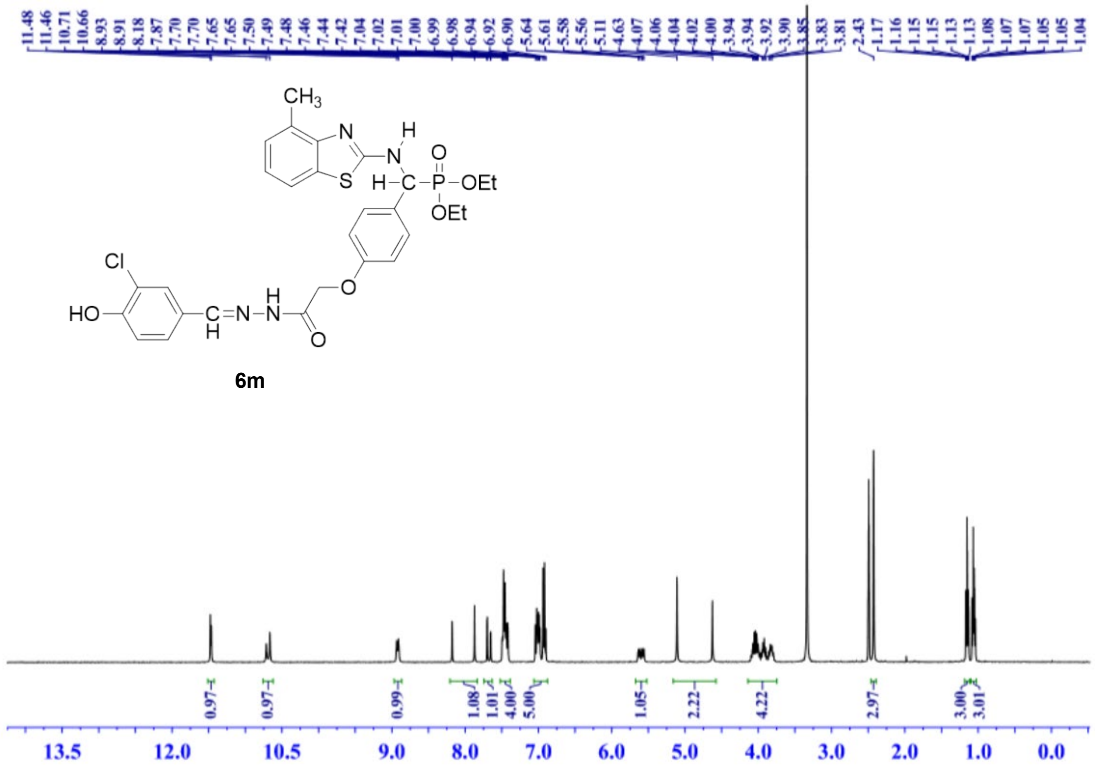

6m

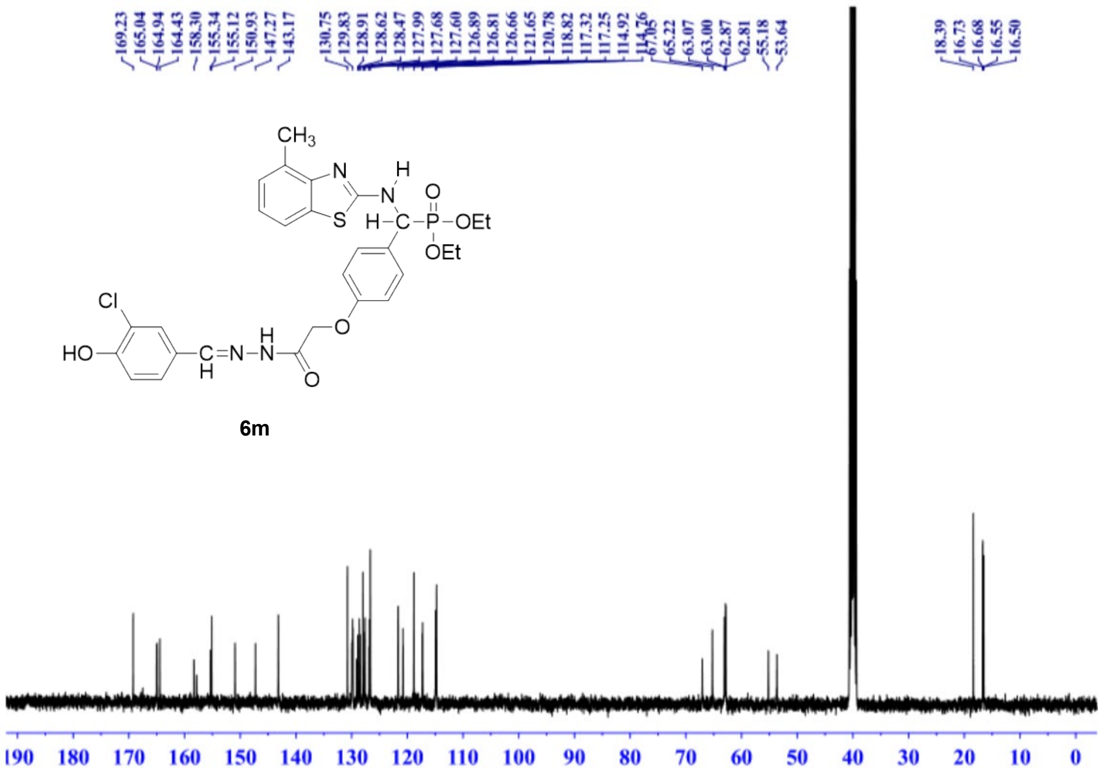

6m
